# Osteoporosis and Normocalcemic Primary Hyperparathyroidism (Conservatively or Surgically Managed)

**DOI:** 10.3390/jcm13216325

**Published:** 2024-10-23

**Authors:** Ana-Maria Gheorghe, Claudiu Nistor, Aurelian-Emil Ranetti, Adrian Ciuche, Mihai-Lucian Ciobica, Mihaela Stanciu, Denisa Tanasescu, Florina Ligia Popa, Mara Carsote

**Affiliations:** 1 PhD Doctoral School, “Carol Davila” University of Medicine and Pharmacy, 0505474 Bucharest, Romania; ana-maria.gheorghe@drd.umfcd.ro; 2 Department of Clinical Endocrinology V, “C.I. Parhon” National Institute of Endocrinology, 011863 Bucharest, Romania; carsote_m@hotmail.com; 3 Department 4—Cardio-Thoracic Pathology, Thoracic Surgery II Discipline, “Carol Davila” University of Medicine and Pharmacy, 0505474 Bucharest, Romania; 4 Thoracic Surgery Department, “Dr. Carol Davila” Central Emergency University Military Hospital, 010825 Bucharest, Romania; 5 Department of Endocrinology, “Carol Davila” University of Medicine and Pharmacy, 020021 Bucharest, Romania; 6 Endocrinology Department, “Dr. Carol Davila” Central Emergency University Military Hospital, 010825 Bucharest, Romania; 7 Department of Internal Medicine and Gastroenterology, “Carol Davila” University of Medicine and Pharmacy, 020021 Bucharest, Romania; lucian.ciobica@umfcd.ro; 8 Department of Internal Medicine I and Rheumatology, “Dr. Carol Davila” Central Military University Emergency Hospital, 010825 Bucharest, Romania; 9 Department of Endocrinology, Faculty of Medicine, “Lucian Blaga” University of Sibiu, 550024 Sibiu, Romania; mihaela.stanciu@ulbsibiu.ro; 10 Medical Clinical Department, Faculty of Medicine, “Lucian Blaga” University of Sibiu, 550169 Sibiu, Romania; denisa.tanasescu@ulbsibiu.ro; 11 Department of Physical Medicine and Rehabilitation, Faculty of Medicine, “Lucian Blaga” University of Sibiu, 550024 Sibiu, Romania; florina-ligia.popa@ulbsibiu.ro

**Keywords:** parathyroid tumor, calcium, PTH, vitamin D, surgery, metabolism, DXA, TBS, parathyroidectomy, fracture

## Abstract

Asymptomatic primary hyperparathyroidism (PHPT) involves 80–90% of the parathyroid tumor-associated cases of PHPT in the modern medical era, while normocalcemic PHPT (NPHPT) has a prevalence of 0.1–11%. We aimed to analyze the bone status and mineral metabolism in NPHPT amid conservative or surgical management. In this narrative review, we searched PubMed (between January 2020 and July 2024) via different keywords. Fourteen studies from the final analysis (388 patients with NPHPT; 1188 with PHPT; and 803 controls) showed that mean serum calcium levels varied between 2.57 and 2.26 mmol/L in NPHPT. Ten studies identified a similar 24 h urinary calcium in NPHPT versus hypercalcemic PHPT (HPHPT). Except for one study, a mandatory vitamin D analysis was performed, but the 25-hydroxyvitamin D cut-offs varied. Osteoporosis (n = 6 studies; N = 172 with NPHPT) was confirmed in 41.7–100% of NPHPT subjects. In surgery candidates, this rate might be overestimated. A DXA analysis was performed in eight studies (235 subjects with NPHPT, and 455 patients with HPHPT); two studies identified a lower BMD in HPHPT < NPHPT, but the results were not homogenous. A single study analyzed the TBS and found similar results in NPHPT. The prevalence of fractures (n = 9) varied between 7.4% and 42.8% in NPHPT. Bone turnover markers (N = 262 patients, n = 8 studies) showed lower bone formation markers in NPHPT versus PHPT (n = 3). Two studies analyzed the BMD and bone turnover markers following parathyroidectomy (161 patients, including 30 patients with NPHPT; mean ages over 60 years). To conclude, given the wide spectrum of complications associated with PHPT, an early diagnosis and proper management is essential. A more extensive screening in patients with osteoporosis and kidney stones might lead to the discovery of NPHPT, a more recently described form of PHPT. While it is still unclear whether NPHPT is an early stage of HPHPT or a separate entity, recent findings show similar osteoporosis and fracture occurrence, and an improvement in bone metabolism, following parathyroidectomy. More extensive prospective studies are crucial to understand the natural course of the disease, to reach a consensus regarding parathyroidectomy indications and surgery candidates’ selection, and to ensure proper personalized management for these patients. With the evolving diagnosis methods, PHPT has become a condition with a changing clinical presentation, which now requires modern evaluation and treatment approaches.

## 1. Introduction

Parathyroid tumors have a large clinical and biochemical panel, with primary hyperparathyroidism (PHPT) being the most commonly associated endocrine disturbance, a condition caused by an excessive tumor-related parathormone (PTH) with phenotypes that range from the classical (symptomatic, traditional) form, which is rarely encountered currently, to the more recently defined normocalcemic primary hyperparathyroidism (NPHPT) [1,2]. Overall, asymptomatic types involve 80–90% of the parathyroid tumor-associated cases of PHPT in the modern medical era, and the values of serum calcium can be elevated or normal in these patients [3,4].

The normocalcemic category of PHPT was first described two decades ago in subjects who underwent extensive investigations for osteoporosis, low-trauma fractures, and/or low bone mass, etc. [5,6]. It was classified as a distinct entity at the Third International Workshop on the Management of Asymptomatic Primary Hyperparathyroidism in 2008 [7]. NPHPT has been increasingly found both in symptomatic and asymptomatic patients who were confirmed with a high serum PTH [8]. The current prevalence varies between 0.1% and 11%, noting the fact that this rate depends on inconsistent diagnosis criteria, different 25-hydroxyvitamin D cut-offs, and multiple methods of identifying new cases of blood PTH excess [9,10,11,12].

According to the current guidelines, NPHPT is characterized by elevated parathormone levels with normal albumin-adjusted total and ionized serum calcium. These findings need to appear in at least two assays during three to six months [13]. No specification is included with respect to the serum phosphorus (as similarly seen in hypercalcemic type) [13,14]. In addition, at the moment of the diagnosis, secondary causes of hyperparathyroidism such as vitamin D deficiency, chronic kidney disease, and (non-parathyroid tumor-related) hypercalciuria should be ruled out [13,14,15], while some medications such as citrate, foscarnet, EDTA, cisplatin, bisphosphonates, and denosumab may also cause secondary hyperparathyroidism (potentially via iatrogenic hypocalcemia); thus, they might interfere with the recognition of NPHPT [16,17,18]. Furthermore, malabsorption also needs to be excluded in order to achieve an adequate confirmation of the mentioned parathyroid anomaly [19].

**Figure 1:**
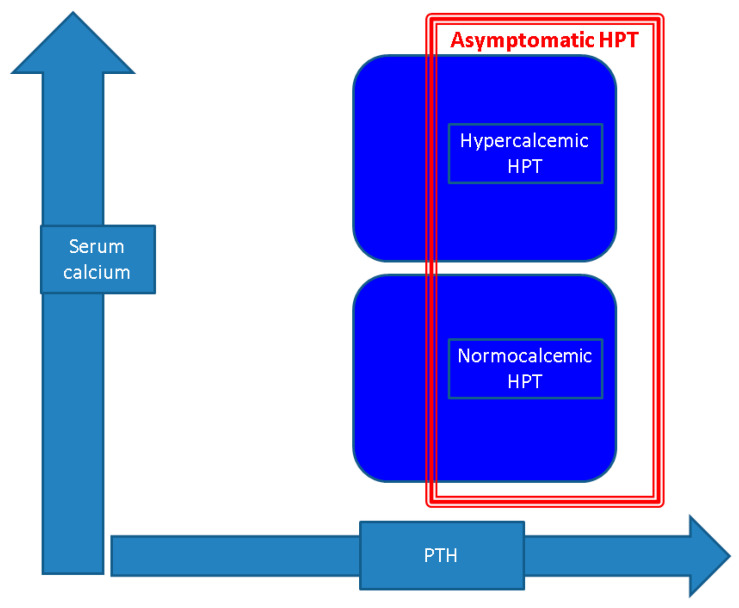
Insights into parathyroid tumor-related PTH excess: hypercalcemic and normocalcemic PHPT both typically show an asymptomatic presentation (which accounts for 80–90% currently); however, not all patients with normocalcemic variant are completely asymptomatic and awareness is needed under this specific circumstance, particularly with respect to the long-term case management that is mandatory despite normal serum calcium levels [3,4,8,13].

Over the years and currently, multiple controversies were pinpointed in relation to the concept of NPHPT, including its biological confirmation amid daily practice, the spectrum of complications at first diagnosis and during long standing follow-up, and the multimodal management, particularly the decision of parathyroidectomy versus a conservative approach. Also, the timing of re-assessment in terms of blood assays of the mineral metabolism; the exploration of potential complications such as bone, renal, and cardiac; and the usefulness of 24 h urinary calcium in patients who did not primarily undergo a parathyroidectomy have been discussed [10,13,20]. Generally, patients diagnosed with PHPT frequently present with osteoporosis and/or urolithiasis due to the complications of long-term high PTH (with or without elevated serum ionized calcium). This may be a selection bias considering that most cases are confirmed with NPHPT due to the screening of serum calcium and parathormone in individuals suffering from osteoporosis and/or kidney stones [10,20,21]. There are, however, studies that identified subjects with normocalcemic variant in the general population without skeleton or renal anomalies of any type [22,23]. Therefore, the overall clinical picture might lack accuracy since it embraces a mostly heterogeneous spectrum with multidisciplinary consequences [10,22,23]. Additionally, studies based on a surgical and/or imaging perspective reported a higher rate of a multi-glandular involvement versus single parathyroid tumor in normocalcemic subjects compared with hypercalcemic patients who were biologically confirmed with PHPT, but not all authors agree [10,24] (Figure 1).

A crucial aspect that needs further elucidation is the natural course of NPHPT. Given these heterogeneous data we have so far, inclusion biases (as prior mentioned), possible erroneous classifications, the spontaneous history, and the extent of the multi-layered complications are not entirely clear at this point [25,26]. Moreover, the rate of the switch from normocalcemic to hypercalcemic PHPT (that might initially present an increased PTH level, but with a normal calcemic blood profile) is low [27].

Overall, NPHPT is considered a less severe form of tumor-related PHPT. However, complications associated with this form of hyperparathyroidism are not uncommon, hence the importance of addressing this topic [27,28]. Apart from kidney and bone afflictions, subjects with normocalcemic variant may suffer from cardiovascular and metabolic complications, including hypertension and increased fasting glucose or even type 2 diabetes mellitus, and all of these should be taken into consideration and assessed in daily practice. An elevated serum PTH might cause complications at the clinical and subclinical level, even in patients with normal serum calcemic values, with anomalies including endothelial anomalies, vascular rigidity, high blood pressure, coronary disease, atherosclerosis, etc. In NPHPT, the exact level of calcium-PTH impact and the exact extent of their post-operatory improvement are still open issues, but long-term surveillance is mandatory in patients who are conservatively approached. The pitfalls of defining the true impact of these associated ailments might be overcome by a personalized approach, but, on a larger scale, prospective studies are still needed [29,30,31,32,33,34].

### Objective

We aimed to analyze the most recent data with respect to the bone and mineral metabolism profile in patients diagnosed with NPHPT amid conservative or surgical management. The importance of the topic relates to the multiple anomalies that have been described in this field, including at skeletal level, and the fact that early recognition might help the overall outcome amid surgical correction of the parathyroid condition; modern medicine allows for more frequent diagnoses of NPHPT, hence the importance of multimodal strategies.

## 2. Methods

The design was a narrative review. We searched PubMed papers published between January 2020 and July 2024 via using the following keywords: “normocalcemic primary hyperparathyroidism” alone and in combination with “bone”, “osteoporosis”, “osteopenia”, “fracture”, “bone turnover markers”, “TBS”, “bone geometry”, and “DXA”. We included the studies that provided a clear analysis with respect to serum calcium, phosphorus, 24 h urinary calcium, PTH, vitamin D assays, DXA, bone turnover markers, osteoporotic fractures, cross-sectional or longitudinal cohorts, and surgically or conservatively treated patients. We excluded case reports, case series, reviews, editorials, animal studies, pediatric cohorts, PHPT in pregnancy, and non-English papers.

**Figure 2:**
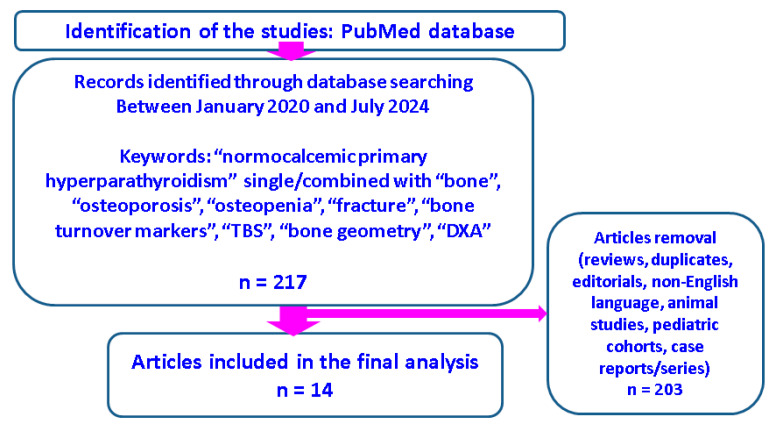
Chart diagram of search.

Out of the initial 217 papers revealed by the search, 14 original studies provided a clinically relevant analysis regarding the bone metabolism in terms of osteoporosis, osteoporotic fractures, bone turnover markers, and DXA findings (Figure 2).

## 3. Results: Studies-Focused Analysis in NPHPT

The fourteen studies included a total number of 388 patients with NPHPT, 1188 subjects confirmed with PHPT, and 803 controls (non-PHPT) [35,36,37,38,39,40,41,42,43,44,45,46,47,48] (Table 1 and Table S1).

The inclusion criteria and criteria that have been used to define NPHPT [35,36,37,38,39,40,41,42,43,44,45,46,47,48] varied greatly among these studies published during the latest five years, while only three studies [40,42,46] took into consideration all criteria recommended by the 2022 guidelines [13]. Two more studies define NPHPT based on high PTH levels, normal albumin-adjusted calcium levels, and normal ionized calcium levels, but did not take into consideration multiple other determinations [43,45]. Almost all studies excluded secondary causes of high PTH. However, Halimi et al. [35] diagnosed PHPT based on a calcium load test [35], which is not a standard requirement according to the guidelines for diagnosis in daily practice [13], and Choi et al. [41] did not provide data on whether vitamin D deficit was excluded, noting that this represents a major differential diagnosis in NPHPT confirmation [41]. Notably, some studies included patients with elevated ionized calcium in the normocalcemic group [39,41], which is not currently recommended for NPHPT diagnosis [13]. Moreover, there might be a selection bias in the studies (n = 5) that included subjects with indications for parathyroidectomy (surgery candidates), which may reflect a more advanced disease stage and thus a potentially more severe bone involvement [37,39,41,42,43].

### 3.1. Analysis of the Serum Calcium, Phosphorus, PTH, and 24 h Urinary Calcium at NPHPT Diagnosis

In subjects with NPHPT, mean serum calcium levels varied between 2.57 mmol/L [36] and 2.26 mmol/L [44], while in hypercalcemic PHPT, they varied between 2.85 mmol/L [36] and 2.62 mmol/L [45]. Mean ionized calcium values were reported in seven studies [35,39,40,41,42,43,46] and varied between 1.19 mmol/L [43,46] and 1.34 mmol/L [39] in subjects with NPHPT, while in those with the hypercalcemic variant, the average ionized calcium ranged between 1.2 mmol/L [40] and 1.45 mmol/L [41]. All studies found statistically significant differences between normocalcemic and hypercalcemic PHPT as expected [35,36,37,38,39,40,41,42,43,44,45,46,47,48].

Moreover, the average parathormone levels varied; for instance, in normocalcemic subjects, the PTH was between 84.75 pg/mL [48] and 149.9 pg/mL [43], while in hypercalcemic patients, the mean PTH values ranged between 93 pg/mL [45] and 212.7 pg/mL [41]. Most studies found higher PTH values in subjects with hypercalcemic PHPT compared to NPHPT [36,38,39,41,42,43,47], while two studies found opposite results, with higher PTH levels in NPHPT [40,45], and another two studies found no statistically significant differences in terms of PTH levels between the two subtypes of PHPT [46,48].

Ten studies [35,36,37,38,39,41,42,43,46,48] reported an analysis of the 24 h (daily) urinary calcium, which is not a standard requirement for the diagnosis of PHPT [49]. Mean (±SD)/median (interquartile interval) values of this parameter ranged between 136.31 ± 90.66 mg/24 h [48] and 315.7 ± 169.3 mg/24 h [41] in NPHPT and between 230.4 (184.3–262.11) mg/24 h [39] and 324.3 ± 178.2 mg/24 h [41] in hypercalcemic PHPT. Most studies did not find statistically significant differences between the normocalcemic and hypercalcemic variants of PHPT [35,41,42,43,48]. On the other hand, three studies reported higher 24 h urinary calcium levels in hypercalcemic patients compared to normocalcemic subjects [36,39,46]. In addition, Chertok Shacham et al. [38] found a higher calcium-to-creatinine ratio in patients with hypercalcemic PHPT compared to NPHPT and controls (0.25 ± 0.19 versus 0.14 ± 0.1 versus 0.15 ± 0.08, *p* = 0.01) [38].

Then other studies provided a serum phosphorus-based analysis [35,36,38,39,40,41,45,46,47,48] and found higher values in individuals with NPHPT versus hypercalcemic PHPT in seven out the ten cohorts [36,38,39,40,41,46,47], while three studies revealed similar levels in the two subgroups [35,45,48]. The highest average serum phosphorus levels in NPHPT were 1.14 ± 0.13 mmol/L (mean age of 65.77 years) [48], while the lowest concentration was 0.8 (0.69–0.85) mmol/L (N = 35 subjects with NPHPT) [35]. Patients with hypercalcemic PHPT had mean phosphate levels between 0.77 (0.65–0.85) mmol/L [35] and 1.06 ± 0.3 mmol/L [48] (Table 2).

### 3.2. Vitamin D Status: Analysis of 25-Hydroxyvitamin D Levels in Patients with NPHPT

Except for one study [38], a mandatory vitamin D analysis was provided amid the mentioned studies [35,36,37,39,40,41,42,43,44,45,46,47,48]. The threshold for vitamin D levels (in terms of 25-hydroxyvitamin D assays) varied. Four studies used the 20 ng/mL (50 nmol/L) lower limit [36,44,45,47], one cohort defined vitamin D deficiency in subjects with values below 24 ng/mL (60 nmol/L) [38], while six other studies applied the cut-off of 30 ng/mL (75 nmol/L) [39,40,42,43,46,48]. Notably, three studies did not provide data regarding vitamin D across the inclusion criteria [35,37,41]. Choi et al. [41] found a mean 25-hydroxyvitamin D of 20.1 ± 8.6 ng/mL in normocalcemic patients [41]. This suggested that individuals with hypovitaminosis D might have been included in the normocalcemic group.

In patients with NPHPT, the mean/median 25-hydroxyvitamin varied between 25.16 (9.4) ng/mL [47] and 35.5 (32.25–41.0) ng/mL [39], while in patients with hypercalcemic PHPT, the mean 25-hydroxyvitamin D varied between 20.1 (13.1–26.6) ng/mL [36] and 37.1 ± 15.4 ng/mL [45]. Six studies found higher vitamin D levels in subjects with NPHPT compared to hypercalcemic patients [35,36,39,40,42,43]. The other seven studies found no statistically significant difference with respect to 25-hydroxivitamin D between sub-groups [38,41,44,45,46,47,48]. Of note, the normocalcemic patients included in the study conducted by Tabacco et al. [40] were under therapy with oral cholecalciferol in order to have normal vitamin D levels [40]. Halimi et al. [35] also analyzed the levels of 1,25-dihydroxyvitamin D, but found no difference between normocalcemic and hypercalcemic patients: 77 (60–96) ng/mL versus 80 (62–101.5) ng/mL (*p* = 0.81) [35] (Table 3).

### 3.3. Prevalence of Osteoporosis/Osteopenia Among Subjects with NPHPT

The presence of osteoporosis and osteopenia was provided in six studies across the entire sample-focused analysis [36,37,39,41,45,48]. It is noteworthy that among these, one study from 2023 had osteoporosis, defined as osteoporotic fracture and/or T-score lower than −2.5 SD at central DXA (Dual-Energy X-Ray Absorptiometry) as an inclusion criteria for the enrolled patients [39], while three other studies investigated subjects with an indication for parathyroidectomy (surgery candidates) [37,41,45]. In surgery candidates, the prevalence of osteoporosis might be overestimated, considering that osteoporosis is one of the parathyroidectomy indications [13].

These studies [36,37,39,41,45,48] included a total of 172 (151 females) patients diagnosed with NPHPT with mean (±standard deviation)/median (interquartile interval) ages between 54.2 ± 12.1 years [41] and 69 (51–85) years [39], and 832 (663 females) with hypercalcemic PHPT with mean/median ages between 57.71 ± 13.24 years [48] and 69 (26–84) years [39].

Additionally, two other studies included subjects with an indication for parathyroidectomy [42,43], one study included patients with osteoporosis or PHPT [38], and another study included individuals who were referred for bone mineral density (BMD) assessments at a specialized center [47], but did not provide data regarding the prevalence of osteoporosis among the patients with NPHPT. The prevalence of osteoporosis varied between 41.7% [36] and 100% [45], depending on the study design and studied population. Half of these studies reporting data about osteoporosis prevalence (n = 3) did not find statistically significant differences in terms of osteoporosis prevalence between hypercalcemic and normocalcemic variants [36,37,48]. For instance, Yankova et al. [36] reported a similar prevalence of osteoporosis between hypercalcemic and normocalcemic PHPT (44.7% versus 41.7%, *p* = 0.575) [36], while Armstrong et al. [37] found that 58.8% of the subjects confirmed with NPHPT had osteoporosis, compared to 37.7% of the hypercalcemic subgroup (*p* = 0.19) [37]. Voss et al. [48] identified a prevalence of 57.1% (NPHPT) versus 53.8% in hypercalcemic PHPT (*p* = 0.848) [48].

Three studies found statistically significant differences in patients with NPHPT versus hypercalcemic variant [39,41,45] in terms of identifying a higher rate of osteoporosis in NPHPT [39,41,45] and a lower prevalence of osteopenia in NPHPT [41]. For example, a longitudinal retrospective cohort study on 109 patients with PHPT and osteoporosis who underwent parathyroidectomy analyzed the prevalence of T-score ≤ −2.5 and reported that more patients with NPHPT and normal ionized calcium were affected than seen in NPHPT with increased serum ionized calcium in hypercalcemic PHPT (97% versus 77%, respectively, 69%, *p* = 0.03). It is notable, however, that a selection bias came from the fact that all included subjects with osteoporosis were further referred for parathyroidectomy [39]. Statistically significant differences were also reported by Choi et al. [41] in a cross-sectional study on 280 subjects with an indication for parathyroidectomy: a lower prevalence of both osteoporosis and osteopenia in subjects with NPHPT compared to the hypercalcemic variant (osteoporosis: 42.4% versus 50%, osteopenia: 30.3% versus 38.6%, *p* = 0.008) was identified. Moreover, a particular subgroup of the subjects confirmed with NPHPT that presented a normal ionized calcium had a higher prevalence of osteoporosis, but a lower prevalence of osteopenia compared with hypercalcemic (traditional) PHPT (osteoporosis: 57.9% versus 50%, osteopenia: 10.5% versus 38.6%, *p* = 0.012). The rates of osteoporosis/osteopenia in NPHPT with normal versus high ionized calcium were similar [41]. As mentioned, people with elevated ionized calcium were considered to have NPHPT, which is outside the current guidelines [13,50]. Additionally, the presence of osteoporosis and a history of (osteoporotic) fragility fracture represent indications for surgery; thus, a more severe clinical presentation has been selected in this cohort that overestimates the rate of osteoporosis in NPHPT [13,41,50]. Other data come from smaller sized studies, for instance, an observational study (N = 43) from 2020 reported osteoporosis in all subjects with NPHPT (N = 7/7, 100%) and in only a third of the individuals with the hypercalcemic variant (N = 9/29; 31%, *p* = 0.008) [45] (Table 4).

### 3.4. Central DXA-Based BMD and T-Score in NPHPT

A central DXA analysis was performed in eight studies that enrolled 235 subjects with NPHPT, and 455 patients with hypercalcemic PHPT [36,38,40,41,42,43,46,47]. The mean age in NPHPT group varied between 54.6 ± 16.3 years [41] and 59 ± 11.8 years [36], while in hypercalcemic cohorts, the highest average age was 69 ± 9.1 years [38]. Control subgroups (non-PHPT) had a mean age between 64.7 ± 7 years [46] and 70 ± 20 years [47]. Two studies [38,41] identified statistically significant differences in lumbar BMD: Chertok Shacham et al. [38] showed a lower T-score in hypercalcemic PHPT versus NPHPT (−1.9 ± 0.9 versus −1.7 ± 1, *p* = 0.04) [38], respectively, and Choi et al. [41] found similar results in a cross-sectional study (N = 280 subjects with indication for parathyroidectomy, including 122 individuals with NPHPT) with an average T-score of −2.4 ± 1.2 versus −2.0 ± 1.3 (*p* = 0.024) [41]. The other five studies that provided a lumbar BMD/T-score analysis showed similar results in NPHPT versus hypercalcemic type [36,40,42,43,46], with regard to the femoral neck and total hip DXA assessment (n = 6 studies) [36,38,40,42,43,46]. Only four studies reported data regarding DXA results at the third distal radius; three of them [36,42,43] confirmed a similar BMD and T-score in NPHPT versus hypercalcemic PHPT; in contrast, a multicenter, cross-sectional study conducted by Palermo et al. [46] found a higher BMD (0.605 ± 0.08 versus 0.563 ± 0.078, *p* < 0.05) and T-score (–1.6 ± 1.2 versus –2.3 ± 1.3, *p* < 0.05) in NPHT versus hypercalcemic PHPT [46] (Table 5).

### 3.5. Bone Quality Assessment: Trabecular Bone Score (TBS) and Bone Strain Index (BSI)

The single study that analyzed the TBS, which we identified according to our methods, had a case–control design; Tabacco et al. [46] reported the TBS in 170 patients with NPHPT, comparing both with subjects with hypercalcemic PHPT and controls (normal PTH). The normocalcemic group had a similar TBS with both the hypercalcemic (1.29 ± 0.14 versus 1.24 ± 0.10, *p* > 0.05) and controls (1.29 ± 0.14 versus 1.3 ± 0.07, *p* > 0.05). However, hypercalcemic subjects had a lower TBS than controls (*p* = 0.009) [46]. The same study also analyzed the BSI, which was the highest in hypercalcemic PHPT, followed by NPHPT, with controls having the lowest value (at all sites). The differences were statistically significant pair-wise between PHPT and controls. In addition, subjects with NPHPT had a statistically significantly lower BSI than hypercalcemic PHPT at femoral neck and total hip. There were no BSI differences between the individuals with NPHPT and controls at any site [46] (Table 6).

### 3.6. Fractures and Normocalcemic PHPT

Data regarding prevalent (low-trauma or osteoporotic) fractures were heterogeneous (n = 9 studies) [36,37,38,39,40,41,44,45,46]. The fractures have been addressed either as prior fractures at any time and type [37,38,41,44], in the latest five years [39], only vertebral fractures [36,45], or specifically fragility fractures [36,45], overall enrolling 297 subjects with NPHPT (out of which 246/297 were females). The prevalence of fractures in NPHPT varied between 7.4% [41] and 42.8% [45]. Only one study provided a fracture risk assessment [46].

Overall, only one cohort identified a statistically significant difference in terms of a higher fracture prevalence (fracture of any type) in NPHPT versus hypercalcemic patients (these data did not undergo paired comparison) [37]. A distinct analysis in osteoporotic fractures was available, as mentioned, in two studies (N = 53 patients). Their prevalence was similar in normocalcemic and hypercalcemic PHPT [36,45]. Vertebral fractures occurred in 20–28% of the NPHPT subjects; a statistically significant lower prevalence of moderate–severe vertebral fractures in NPHPT versus hypercalcemic PHPT was confirmed (5% versus 20.4%, *p* < 0.05) [40,45].

The study by Armstrong et al. [37] had the largest size (N = 421 individuals with PHPT) and included 39 subjects with NPHPT, as well as 42 with normal hormonal PHPT. The authors reported a higher prevalence of fractures among subjects with normocalcemic versus normal hormonal PHPT, compared to hypercalcemic PHPT (12.8% and 24.4% versus 9.8%, *p* = 0.02) [37]. On the other hand, Yankova et al. [36] did not find a statistically significant difference between subjects with normocalcemic and hypercalcemic PHPT in terms of the prevalence of low-energy fractures (8.3% versus 7%, *p* = 0.483) [36]. Additionally, Chertok Shacham et al. [38] (N = 105) found a similar fracture prevalence in subjects with NPHPT versus those with osteoporosis and individuals with hypercalcemic PHPT [38], as observed by Koumakis et al. [39] and Liu et al. [45]. Choi et al. [41] reported similar rates of “bone fractures” in subjects with normocalcemic versus hypercalcemic PHPT (7.4% versus 8.2%, *p* = 0.793) and also in NPHPT with normal ionized calcium when compared to the hypercalcemic subgroup (*p* = 0.885) [41].

Notably, Tabacco et al. [41] reported the prevalence of vertebral fractures in subjects with PHPT in a case–control study on 170 subjects. Even though there was no difference in terms of the vertebral fracture prevalence between subjects with NPHPT and hypercalcemic PHPT or controls, the prevalence of moderate–severe vertebral fractures was four times lower in NPHPT versus hypercalcemic PHPT (5% versus 20.4%, *p* < 0.05) [41]. With regard to the fracture risk assessment, one multicenter, cross-sectional study found that subjects with hypercalcemic PHPT had higher odds of fractures [OR = 5.87 (2.16–17.3)], of moderate–severe fractures [OR = 3.81 (1.15–15.12)], and of developing more than one fracture [relative risk of (RR) of 2.24 (1.22–4.32)] compared to controls, but the same risks were similar between controls and NPHPT [46].

Particularly for the male population, Kontogeorgos et al. [44] performed a prospective cohort study in 750 men who were followed for 21 years. After this period of surveillance (at age 71), 21 (2.8%) of them developed NPHPT, but this subgroup did not demonstrate a higher number of fractures than those with normal calcium and normal parathormone (5% versus 6%, *p* > 0.5) [44].

To summarize, the heterogeneity of the analyzed outcomes, the small number of patients, and the lack of statistical significance in most areas concerning the fractures prevalence suggest that this remains an open matter, rather than a conclusion, indicating that further research is needed (Table 7).

### 3.7. Bone Turnover Markers

Eight studies included data regarding bone turnover markers in NPHPT (N = 262 patients, n = 8 studies) [35,36,38,42,43,46,47]. Three of these studies found lower levels of bone formation markers in normocalcemic compared with hypercalcemic PHPT [36,42,43]; specifically, two cohorts confirmed reduced bone formation markers osteocalcin, P1NP, and the bone resorption marker β-cross-linked telopeptide of type I collagen (β-CTX) in normocalcemic versus hypercalcemic PHPT [42,43]. Osorio-Silla et al. [42] performed a prospective study on 87 subjects with PHPT who were referred for surgery (osteocalcin: 24.4 ± 17.4 versus 37 ± 17.4 ng/mL, *p* = 0.007, P1NP: 55.4 ± 30.2 versus 71.2 ± 30.6 ng/mL, *p* = 0.03, β-CTX: 0.4 ± 0.3 versus 0.7 ± 0.4 ng/mL, *p* = 0.01) [42]. Gomez-Ramirez et al. [43] also identified a lower osteocalcin, P1NP, and β-CTX in normocalcemic versus hypercalcemic PHPT [43]. Out of these eight studies, six provided data with concern to the alkaline phosphatase (total enzyme, specific bone enzyme, or enzyme activity) [35,36,42,43,47]. The study conducted by Yankova et al. [36] was the only study (n = 1/6 studies) reporting a statistically significant difference in terms of a lower serum level in normocalcemic versus hypercalcemic PHPT (*p* = 0.016) [36].

To summarize, these findings suggest that bone turnover, as analyzed by the formation (alkaline phosphatase, osteocalcin, P1NP) and resorption (β-CTX) markers, might be lower in subjects with normocalcemic compared to hypercalcemic patients with PHPT diagnosis [35,36,38,42,43,46,47]. However, as seen in multiple other bone conditions, including bone metabolic diseases or primary/secondary bone malignancies, the bone turnover markers picture suffers a great area of intra- and inter-personal variation; hence, a clear conclusion is hardly applicable on a matter of an individual basis [51,52,53] (Table 8).

### 3.8. The Impact of Parathyroidectomy on the Bone Status in NPHPT

Two studies [42,43] provided data regarding changes in the BMD and bone turnover markers following parathyroidectomy; a total of 161 patients, including 30 patients with NPHPT (mean ages over 60 years) were followed for at least one year after parathyroid surgery that was recommended by an endocrinologist in all cases. Both studies classified NPHPT based on elevated PTH and normal albumin-adjusted serum calcium and ionized calcium on multiple assays, in accordance with the current guidelines released in 2022 by Bilezikian et al. [13]. Regarding the bone turnover markers profile, these studies had different approaches, while Osorio-Silla et al. [42] analyzed the improvement in terms of BMD and bone turnover markers following parathyroidectomy in subjects with hypercalcemic versus normocalcemic variants [42]. Gomez-Ramirez et al. [43] compared the same parameters before and after surgery [43]. Overall, patients with NPHPT seemed to have an improvement in the BMD at femoral neck [42,43].

Osorio-Silla et al. [42] followed patients for 24 months in a prospective study (N = 87 subjects with PHPT, including 16 with NPHPT, who underwent parathyroidectomy). One year after surgery, there was a higher increase in the lumbar BMD in the hypercalcemic versus normocalcemic type (3.6% versus 2.8%); both groups had an increase in the femoral neck BMD, while the BMD at the level of the third distal radius was similar. Of note, after two years, only subjects with hypercalcemic PHPT showed a further improvement in the lumbar BMD (+1.1%, *p* = 0.02). With regard to the bone turnover biomarkers, both groups had them normalized 12 months following the removal of the parathyroid tumor [42]. Gomez-Ramirez et al. [43] compared 88 individuals with hypercalcemic PHPT and 16 with NPHPT who underwent surgery and found no BMD difference between these groups, while pre-operatory statistically significant differences between bone turnover markers shifted into a similar profile after parathyroidectomy [43].

These two studies have some limitations in terms of a small sample size and a relatively short duration of surveillance amid surgery (one to two years after parathyroid tumor removal), while a selection bias comes from the fact that only surgery candidates were enrolled. A larger perspective should include a prevalent and incidental fractures profile and fracture risk assessment. Osorio-Silla et al. [42] did not compare the BMD changes between the groups; therefore, we cannot conclude upon differences between normocalcemic and hypercalcemic patients regarding the true benefit of parathyroidectomy [42]. In contrast, Gomez-Ramirez et al. [43] did not investigate the change in bone parameters in the same group (pre- and post-operatory), but provided data regarding the differences between normocalcemic and hypercalcemic patients following parathyroidectomy [43] (Table 9).

## 4. Discussion

This sample-focused analysis pinpointed across fourteen studies a heterogeneous spectrum of results in the field of NPHPT-related mineral and bone metabolism [35,36,37,38,39,40,41,42,43,44,45,46,47,48]. Generally, it was first hypothesized that NPHPT stands for the early stage of PHPT that later turns into a hypercalcemic presentation; however, this is not the case for all patients [54,55]. Other hypotheses include different possible underlying causes and interplays between aging, menopausal status in females, a partial resistance to PTH in the kidney and bones, polymorphisms of the calcium sensing receptor, persistent low levels of free 25-hydroxivitamin D, or the co-presence of other (non-endocrine) co-morbidities, etc. [54,55,56,57,58,59]. The current guidelines do not recommend parathyroidectomy in cases with the normocalcemic variant due to the inconsistent data we have so far [13]. Further prospective studies are needed to identify the possible benefit of an early intervention on the reduction in fracture risk and even in other cardio-metabolic outcomes.

### 4.1. Definition and Management Pitfalls in NPHPT

When analyzing the serum calcium levels, both albumin-adjusted calcium and ionized calcium need to be measured [13]. Almost half of the total calcium circulates were bound to albumin, while half circulated as hydrated cation, widely named as “ionized calcium” [60]. Assessing albumin and adjusting it, especially for levels below 4 g/dL, is needed in order to avoid an underestimation of hypoalbuminemia [13]. Some authors suggested that the equation used to determine albumin-adjusted calcium should be determined by each laboratory, as it may differ depending on the technique and method used [61,62,63]. Considering pH influence on calcium determination and the variability of albumin-adjusting formulas, recent studies suggested the use of ionized calcium for establishing the calcium status [64]. It is considered a much more accurate reflection of the calcium status, especially at high PTH levels, and determination by an analyzer is preferred [13,64,65]. Lately, there is also controversy regarding the normal levels of calcium. For instance, Schini et al. [66] suggested that current normal ranges should be updated and proposed age- and sex-dependent levels (upper limits: women of 55–69 years = 2.59 mmol/L, women of 40–55 years = 2.57 mmol/L, men = 2.55 mmol/L) based on findings from a large cross-sectional study (N = 502,524 subjects) [66]. Multiple assays are also important due to the variability of calcium levels and the physiological fluctuations of PTH [67,68]. Since there is a potential of an intermittent normocalcemia and hypercalcemia, Schini et al. [66] proposed a least significant change for albumin-adjusted calcium of 0.25 mmol/L (1 mg/dL); this might come as a threshold to establish whether these calcium variations indicate a change in the overall presentation [66]. Another diagnostic issue is the lack of concordance between different intact parathormone assays that might result in inclusion biases [69,70].

In addition to this debate concerning calcium levels, albumin-adjusting formulas, and assays differences, the heterogeneity of the recent definition criteria for NPHPT adds another limitation to the data quality. Another aspect of the diagnosis pitfalls, apart from finding normal albumin-adjusted serum calcium and ionized calcium associated with high PTH levels, is the exclusion of secondary causes of hyperparathyroidism, as mentioned [13]. Kidney function is mandatory to be evaluated in order to exclude secondary hyperparathyroidism in chronic kidney disease (stages higher than G3 that correspond to a glomerular filtration rate of <60 mL/min/1.73 m^2^ are usually associated with a secondary increase in PTH [71,72]. Last, but not least, the conversion to hypercalcemic forms remains another highly debated question, considering that, after longer follow-ups (more than two to four years), some, but not all, patients progress to hypercalcemia [73].

### 4.2. Vitamin D Interplay in NPHPT

Vitamin D, a secosteroid with multiple metabolites, plays a part in different metabolic and biochemical pathways including calcium homeostasis, bone metabolism, and PTH secretion [74]. 1,25-dihydroxyvitamin D and 25-hydroxyvitamin D bind to the vitamin D receptor (VDR), which is present in many tissues including bone, kidney, and the parathyroid glands [75,76]. In the parathyroid glands, 1,25-dihydroxyvitamin D suppresses PTH gene expression and decreases parathyroid cell proliferation, leading to a decrease in PTH secretion [77]. Low levels of vitamin D therefore lead to the development of secondary hyperparathyroidism (as mentioned) with elevated PTH secretion [78]. Levels of 20 to 30 ng/mL (50 to 75 nmol/L) are associated with a PTH increase; patients with <30 ng/mL should be treated with cholecalciferol and be re-assessed for PTH after 3–6 months [79]. Recent data revealed variable cut-offs that add to the heterogeneous spectrum of defining NPHPT [35,36,37,39,40,41,42,43,44,45,46,47,48].

Some authors also suggested a connection between free vitamin D and PHPT, noting the correlation between higher free 1,25-dihydroxyvitamin D and increased PTH in these patients [80]. Further research is needed to explore this relationship in normocalcemic patients as well. It should be taken into consideration that sometimes secondary hyperparathyroidism (due to vitamin D deficit) may not be a solitary condition and may exert a cumulative effect with PHPT, thus causing a more severe presentation [81,82]. In order to exclude this scenario of overlap, surveillance after at least three months of vitamin D supplementation is suggested; but, in some cases, the normalization of PTH may take up to a year [83,84]. Sometimes, vitamin D correction may reveal a true hypercalcemic PHPT [84]. Whether the disease is more severe in patients with cumulative causes of high PTH is still an open matter.

Notably, with regard to mineral metabolism, we should also mention, not only the crossroads with calcium, PTH, and vitamin assays in NPHPT, but also the phosphate assessment, which is not always taken into account during the management of PHPT [85]. Recent data suggested that low phosphate levels may predict a recommendation of further parathyroidectomy [86]. Although rare, hypophosphatemia might be part of the clinical picture in NPHPT in rare cases [87].

### 4.3. NPHPT Versus Hypercalcemic (Typical) PHPT: Sample-Focused Results in the Bone Profile

Data regarding the impact of NPHPT on bone from the most recent studies indicate alterations in bone metabolism, as mentioned above [35,36,37,38,39,40,41,42,43,44,45,46,47,48]. Osteoporosis was a common finding in normocalcemic patients, similar to hypercalcemic subjects [36], or was even more frequent [41,45]. Other findings were contradictory. For instance, normocalcemic individuals had a lower T-score compared to hypercalcemic patients, but had a similar BMD [38] in one study, while another cohort pinpointed a higher T-score in NPHPT [41]. The TBS was similar, as was the lumbar BSI. The femoral and total hip BSI, however, suggested less strain in normocalcemic versus hypercalcemic patients across the single study we could identify according to our methods [40]. Fragility fractures also had a similar prevalence in normocalcemic and hypercalcemic subgroups [36,45], as similarly shown for bone fractures in general [41], while another cohort showed that moderate–severe vertebral fractures were less frequent in NPHPT [40]. Some studies reported that patients with NPHPT had lower bone turnover markers [42,43], and others reported similar levels compared to hypercalcemic patients [35,36]. Considering the small number of studies, it is still rather difficult to assess the true impact of NPHPT on bone compared to traditional PHPT. However, NPHPT appears to be associated with osteoporosis and fractures as well.

Even though the diagnosis of osteoporosis in the absence of fragility fractures is based on the BMD at a central DXA scan, the BMD might not accurately predict the fracture risk [88]. In patients with NPHPT, one study reported both a similar BMD (lumbar, hip, and third distal radius) and fragility fractures prevalence compared to hypercalcemic patients [36]. On the other hand, one study found discordant findings such as a fracture prevalence of normocalcemic patients lower than in PHPT, but higher than in controls, which was not reflected by the BMD analysis in the three groups [46].

In patients with PHPT, the osteoporotic fracture risk is increased [89,90]; so far, this is not a distinct input in FRAX^®^, but it is taken into consideration as a secondary osteoporosis variable. However, secondary osteoporosis influences the results only in patients without BMD measurements [89,91,92,93]. Data regarding fracture risk in NPHPT are heterogeneous. A similar fracture risk between NPHPT and controls was found, with an odd ratio of 1.32 (0.48–3.72) in one study [46]. To what extent NPHPT influences fracture risk assessment remains an open question.

According to earlier studies, it was believed that PHPT mostly affects the cortical bone, while sparing trabecular bone. However, recent data reported alterations of the (trabecular) bone microarchitecture as part of the extensive changes in the bone metabolism [94]. The TBS, high-resolution peripheral quantitative computed tomography (HR-pQCT), and even invasive methods such as bone biopsy might reflect the trabecular bone anomalies in PHPT [95]. The TBS (as calculated based on lumbar spine DXA), a non-invasive method that strongly reflects the microarchitecture, might be helpful in PHPT (low TBS) as seen in type 2 diabetes mellitus and glucocorticoid osteoporosis [96,97,98]. Some studies did not find an association between TBS and fracture prevalence in PHPT. For instance, Jones et al. [99] reported a TBS ≤ 1.2 in 57.4% of the patients with PHPT, while only 20% of them had osteoporosis according to the BMD at DXA [99]. Arboiro-Pinel et al. [100] found a reduced TBS in PHPT, but no difference in terms of the TBS between patients with fractures and those without [100]. TBS data in NPHPT remain scarce. Tabacco et al. [40] found a similar TBS in NPHPT and hypercalcemic PHPT compared to controls (but a lower TBS in PHPT than controls) [40]. To what extent low TBS scores translate into fracture discriminator in PHPT/NPHPT remains an open issue.

Notably, HR-pQCT, a non-invasive three-dimensional imaging method that may be used to assess cortical and trabecular bone, is not widely available yet [101]. Recent data provided by HR-pQC investigations revealed alterations of both the trabecular and cortical bone microarchitecture at the radius and tibia in PHPT [102]. Another recent tool for bone evaluation is the BSI, a parameter of deformation calculated based on lumbar or femoral DXA scans, which reflects the bone strength [103]. igh BSI has been associated with an elevated fragility fracture risk, and it was also linked to an increased re-fracture risk in subjects with osteoporosis and previous fractures [104,105]. In subjects with PHPT, a study on 50 patients and 100 controls reported a higher BSI at lumbar spine, femoral neck, and total hip. Moreover, a lumbar BSI higher than 2.2 independently predicted vertebral fractures, with the odds ratio adjusted for sex and age being 6.887 (1.628–29.138; *p* = 0.009) or, with multiple adjustments, 15.120 (1.059–215.786; *p* = 0.045) [106]. As specified, we identified only one recent study in the lumber BSI amid NPHPT confirmation with similar values in NPHPT versus controls. Moreover, the BSI was lower than in subjects with the hypercalcemic variant at femoral neck and total hip. All these findings suggested that bone strength might be less affected in NPHPT, but larger, prospective studies are needed [40].

Bone turnover markers reflect bone remodeling determined by the interaction between osteoblasts, osteoclasts, and osteocytes; the osteoclasts-derivate resorption is reflected by β-CTX, while osteoblasts-connected bone formation is pinpointed by assessing bone alkaline phosphatase, osteocalcin, and procollagen I N-propeptide (P1NP) [107]. They are widely used, especially in postmenopausal osteoporosis under specific medication [108].

In PHPT, the intense bone remodeling generates elevated levels of both resorption and formation bone turnover markers [109]; bone turnover markers may also reflect the disease activity [110], while a decrease in bone turnover markers was found post-operatively [111,112]. Some data suggested a lower level in NPHPT compared to hypercalcemic patients [113].

### 4.4. The Impact of Parathyroidectomy on Bone Status

In patients with PHPT, parathyroidectomy leads to an improvement in the BMD, especially in more advanced forms of the disease [114,115], while the post-operatory fracture risk reduction is less clear [13,116]. Bone data regarding post-surgery status in NPHPT are scarce. Some authors suggested that parathyroidectomy should be taken into account by a multidisciplinary team in NPHT [117]. Older studies provided heterogeneous findings in post-parathyroidectomy BMD amid NPHPT confirmation. Koumakis et al. [118] found an increase in the lumbar BMD (+2.3 ± 5%; *p* = 0.016) and hip BMD (+1.9 ± 5.7%; *p* = 0.048) in NPHPT similar to the increase in hypercalcemic PHPT (*p* > 0.1) [118]. Similar results were reported in another longitudinal cohort, with an increase in the BMD following surgery in 46% of the normocalcemic patients and a higher increase at the level of spine and hip [119]. Other data suggested no post-surgery BMD improvement in NPHPT [120]; Sho et al. [121] found a BMD increase at the site with the lowest preoperative T-score, but only in subjects with normalized post-operative PTH (+5.6%; *p* = 0.006), suggesting that the BMD elevation might be dependent on the surgical cure [121]. Currently, parathyroidectomy is part of the minimal invasive endocrine surgery (as seen for thyroid or adrenal glands), and the post-operatory expected outcomes are very good [13,122], Yet, according to our 5-year search, we only found two studies to connect the bone status with the surgical act in NPHPT [42,43]. Whether a potential influence due to the COVID-19 pandemic should be taken into consideration, as seen in other surgical domains with respect to the parathyroid surgery access, is debatable [123,124,125,126]. We noticed a BMD increase at the femoral neck and not at the third distal radius in NPHPT [42], while post-parathyroidectomy BMD at all sites did not differ between hypercalcemic and normocalcemic subjects [43]. Overall, the latest findings do not stand against the benefit of parathyroidectomy, but the BMD improvement is not consistently sustained in NPHPT.

Older guidelines offered an algorithmic approach for the management of NPHPT, suggesting annual measurements of calcium and PTH accompanied by bone evaluation via DXA. Surgery was recommended in the case of disease progression with an impact on kidneys (stones) or bone (BMD decrease or incidental fractures) [50]. Current guidelines, however, give no opinion for or against surgical management, suggesting that clinicians may be guided by the criteria used for PHPT [13]. There are no specific medical management-based recommendations for patients with NPHPT in general as opposed to PHPT, whereas medical treatments include medications meant to increase the BDM such as alendronate, denosumab, and vitamin D replacement, and drugs designed to reduce the serum calcium levels such as cinacalcet [127,128,129,130]. Studies regarding the medical treatment of NPHPT are scarce and include few patients. One randomized trial on women with NPHPT (N = 30) reported that those treated with alendronate and cholecalciferol displayed a BMD increase (change in lumbar BMD was +4.7%; *p* = 0.001; change in femoral neck BMD was +2.6%; *p* = 0.001; change in total femur BMD was +4%; *p* = 0.001) and a reduction in bone turnover markers, beta-crosslaps, and osteocalcin (*p* < 0.001), unlike the control group. However, calcium and PTH levels were similar upon this conservative approach [131]. Data concerning denosumab use in NPHPT are limited. For example, Konrade et al. [132] reported one case of a 73-year-old woman with NPHPT and osteoporosis treated with denosumab, who developed hypophosphatemia and a further increase in PTH while maintaining normal calcium levels [132].

### 4.5. Current Limits and Further Expansion

We are aware that the present work is a narrative review that only focused on the bone health amid the confirmation and the management, including surgery, of NPHPT, which might be regarded as a limitation. Larger prospective studies are mandatory to analyze the sub-domains of these skeleton issues. But the panel of NPHPT-related complications may be expanded outside the bone status. Urolithiasis is one of the classical complications of PHPT. Similar to PHPT, patients with NPHPT develop kidney stones with a prevalence of 14% to 18%. The prevalence might however be influenced by the selection bias from diagnosing NPHPT following screening in patients with kidney stones [9,10,133,134].

The current bone topic represents a part of the potential issues in NPHPT. Another important topic (which is currently controversial and widely discussed) involves secondary hypertension, large artery stiffness, and coronary calcifications in PHPT; one patient may present anomalies in the bone and mineral status along with cardio-metabolic co-morbidities [135,136,137]. Metabolic syndrome is also a more common finding compared to in the general population, but a direct link is debatable [138,139]. Parathyroidectomy may lower cardiovascular morbidity and mortality in a group of surgery candidates, but the pathogenic pathways are not yet clear [140,141].

Due to the limited and conflicting data, cardiovascular risk is not part of the surgical indications in PHPT [13]. Even though NPHPT is considered a milder form of disease, there is evidence suggesting that NPHPT may also lead to atherosclerosis [142,143]. Cardiovascular changes might be caused, not only by hypercalcemia, but also by high PTH levels, considering that PTH may induce vasodilation, is associated with a positive ionotropic and chronotopic effect, and promotes hypertrophic growth of the cardiac myocytes [144,145].

## 5. Conclusions

Given the wide spectrum of complications associated with PHPT due to the influence of high calcium and parathyroid hormone levels on bones and kidneys, calcium screening remains the key to an early diagnosis and proper management. The more extensive screenings for underlying pathologies in patients with osteoporosis and kidney stones led to the discovery of normocalcemic primary hyperparathyroidism, a more recently described form of primary hyperparathyroidism. While it is still unclear whether this disease is an early form of primary hyperparathyroidism or a separate entity altogether, recent findings show similar osteoporosis and fracture occurrence, as well as an improvement in bone metabolism following parathyroidectomy, and argue for screening for this disease. More extensive prospective studies are crucial to understand the natural course of this disease, to reach a consensus regarding parathyroidectomy indications and surgery candidates’ selection, and to ensure proper personalized management for these patients. With the evolving diagnosis methods, primary hyperparathyroidism has become a disease with a changing clinical presentation that now requires modern evaluation and treatment approaches.

## Figures and Tables

**Table 1:** Studies in patients with NPHPT that provided data with regard to the bone status and mineral metabolism according to our methods [35,36,37,38,39,40,41,42,43,44,45,46,47,48] (Abbreviations: BMD = bone mineral density; BTM = bone turnover marker; Ca = calcium; eGFR = estimated glomerular filtration rate; F = female; hPHPT = hypercalcemic primary hyperparathyroidism; iCa = ionized calcium; M = male; N = number; nPHPT = normocalcemic primary hyperparathyroidism; nhPHPT = normal hormonal PHPT; PTH = parathormone; PHPT = primary hyperparathyroidism; TBS = trabecular bone score; 25OHD = 25-hydroxyvitamin D; y = year).

First Author, Year of Publication, Reference Number Study Number	Study Design, Studied Population	Criteria Used for NPHPT Selection	Category of Findings Regarding Bone Metabolism (Outcomes)
Halimi 2024 [35] 1.	Retrospective study N = 91 with PHPT and kidney stones and hypercalciuria N1 = 56 with hPHPT N2 = 35 with nPHPT	PHPT: Serum iCa > 1.31 mmol/L and PTH > 30 pg/mL after Ca load test nPHPT: iCa < 1.31 mmol/L pre-test	BTM
Yankova 2024 [36] 2.	Retrospective study N = 316 consecutive patients with PHPT N1 = 266 with hPHPT F:M = 234:32 (Mean age = 59.0 ± 11.8 y) N2 = 48 with nPHPT F:M = 42:6 (Mean age = 56.9 ± 13.4 y)	High PTH and high-normal Ca, at 2 measurements Normal 24 h urinary calcium 25OHD > 20 ng/mL (50 nmol/L) eGFR > 60 mL/min/1.73 m^2^ Without other secondary causes (malabsorption, drugs)	Osteoporosis prevalence Fracture prevalence BMD BTM
Armstrong 2023 [37] 3.	Retrospective study N = 421 with PHPT referred for parathyroidectomy F:M = 307:114 (Mean age = 65.6 ± 12.2 y) N1 = 340 with hPHPT F:M = 244:96 (Mean age = 65.6 ± 12.4 y) N2 = 39 with nPHPT F:M = 32:7 (Mean age = 67.3 ± 9.0 y) N3 = 42 with nhPHPT F:M = 31:11 (Mean age = 64.0 ± 13.3 y)	High PTH and normal serum calcium at ≥2 measurements	Osteoporosis prevalence Fracture prevalence
Chertok Shacham 2023 [38] 4.	Prospective, observational study N = 105 referred for PHPT, osteoporosis, or elevated PTH level with normal serum calcium F:M = 98:7 (Mean age = 69 ± 7.9 y) N1 = 30 with hPHPT F:M = 27:3 (Mean age = 69.3 ± 9.1 y) N2 = 30 with nPHPT F:M = 28:2 (Mean age = 69.7 ± 7.2 y) N3 = 45 with osteoporosis without PHPT F:M = 43:2 (Mean age = 68.4 ± 7.7 y)	High PTH and normal serum-corrected calcium Without secondary causes and 25OHD > 60 nmol/L (24 ng/mL)	Fracture prevalence BMD and T-scores BTM
Koumakis 2023 [39] 5.	Longitudinal retrospective cohort study N = 109 with PHPT and osteoporosis who underwent parathyroidectomy F:M = 97:12 [Median age = 68 (26–92) y] N1 = 32 with hPHPT F:M = 29:3 [Median age = 69 (26–84) y] N2 = 39 with nPHPT with elevated ionized calcium F:M = 34:5 [Median age = 69 (43–92) y] N3 = 38 with nPHPT with normal ionized calcium F:M = 34:4 [Median age = 65 (51–85) y]	High PTH and normal total calcium Calcium load test: increased ionized calcium with minimal reduction in PTH 25OHD > 30 ng/mL (75 nmol/L) eGFR > 60 mL/min no malabsorption	Osteoporosis prevalence Fracture prevalence BTM
Tabacco 2023 [40] 6.	Case–control study N = 170 with PHPT and controls F:M = 159:11 (Mean age = 64.9 ± 9.3 y) N1 = 50 with hPHPT F:M = 47:3 (Mean age = 65.2 ± 11.6 y) N2 = 40 with nPHPT F:M = 37:3 (Mean age = 63.4 ± 9.0 y) N3 = 80 age-matched controls F:M = 75:5 (Mean age = 65.4 ± 7.8 y)	High PTH and normal albumin-corrected and ionized serum calcium at ≥2 determinations Without secondary causes 25OHD > 30 ng/mL (75 nmol/L)	Fracture prevalence BMD TBS and bone strain index
Choi 2022 [41] 7.	Cross-sectional study N = 280 (with indication for parathyroidectomy) N1 = 158 with hPHPT F:M = 120:38 (Mean age = 59.3 ± 14.0 y) N2 = 122 with nPHPT F:M = 105:17 (Mean age = 54.3 ± 13.1 y) N3 = 95 with elevated ionized Ca in nPHPT F:M = 82:13 (Mean age = 54.2 ± 12.1 y) N4 = 27 with normal ionized Ca in nPHPT F:M = 23:4 (Mean age = 54.6 ± 16.3 y)	High PTH and normal corrected serum calcium with normal or high ionized calcium	Osteoporosis prevalence Fracture prevalence T-scores
Osorio-Silla 2022 [42] 8.	Prospective study N = 87 with PHPT referred for parathyroidectomy (at the indication of an endocrinologist) (30 patients were lost to follow-up) F:M = 68:19 N1 = 71 with hPHPT (28 patients were lost to follow-up) F:M = 55:16 (Mean age = 61.4 ± 11 y) N2 = 16 with nPHPT (2 lost to follow-up) F:M = 13:3 (Mean age = 61.6 ± 11 y)	High PTH and normal albumin-adjusted calcium and ionized calcium at ≥3 measurements No secondary causes 25OH > 30 ng/mL (75 nmol/L))	BMD and T-scores BTM BMD and BTM following parathyroidectomy
Gomez-Ramírez 2020 [43] 9.	Comparative prospective study N = 104 with PHPT who underwent parathyroidectomy N1 = 88 with hPHPT F:M = 68:20 (Mean age = 60.6 ± 11 y) N2 = 16 with nPHPT F:M = 13:3 (Mean age = 60.9 ± 10.4 y)	High PTH and normal ionized and albumin-corrected serum calcium No secondary causes Normal renal function 25OHD > 30 ng/mL (75 nmol/L)	BMD BTM BMD and BTM following parathyroidectomy
Kontogeorgos 2020 [44] 10.	Prospective cohort study N = 750 men (population sample) Age = 50 y N1 = 3 with hPHPT N2 = 21 with nPHPT N3 = 3 with secondary HPT N4 = 680 with normal PTH N5 = 312 with normal calcium, PTH and vitamin D	High PTH and normal albumin-corrected calcium 25OHD ≥ 50 nmol/L (20 ng/mL)	Fracture prevalence—in men
Liu 2020 [45] 11.	Observational study N = 43 women with PHPT N1 = 29 with hPHPT (Mean age = 6.9 ± 7.3 y) N2 = 7 with nPHPT (Mean age = 66.7 ± 6.2 y) N3 = 7 controls (Mean age = 61.6 ± 5.6 y)	High PTH and normal ionized and albumin-corrected serum calcium No secondary causes Normal renal function 25OHD ≥ 20 ng/mL (50 nmol/L)	Osteoporosis prevalence Fracture prevalence
Palermo 2020 [46] 12.	Multicenter cross-sectional N = 127 with PHPT and controls F:M = 115:12 (Mean age = 64.1 ± 9.6 y) N1 = 41 with hPHPT F:M = 38:3 (Mean age = 63.9 ± 12 y) N2 = 47 with nPHPT F:M = 43:4 (Mean age = 63.8 ± 9.3 y) N3 = 39 controls F:M = 35:4 (Mean age = 64.7 ± 7 y)	High PTH and normal albumin-corrected and ionized calcium at ≥2 measurements No secondary causes 25OHD > 30 ng/mL (75 nmol/L)	Fracture prevalence BMD and T-scores BTM
Schini 2020 [47] 13.	Retrospective study N = 6280 referred for BMD measurements, out of which: N1 = 17 with hPHPT F:M = 15:2 (Mean age = 67 ± 6 y) N2 = 11 with nPHPT F:M = 10:1 (Mean age = 68 ± 11 y) N3 = 300 controls F:M = 214:86 (Mean age = 70 ± 20 y)	High PTH and normal albumin-corrected Normal kidney function 25OHD ≥ 50 nmol/L (20 ng/mL)	Z-scores
Voss 2020 [48] 14.	Case–control study N = 40 postmenopausal women with PHPT and controls N1 = 7 with hPHPT (Mean age = 57.71 ± 13.24 y) N2 = 13 with nPHPT (Mean age = 65.77 ± 12.74 y) N3 = 7 controls for N1 (Mean age = 57.00 ± 13.10 y) N4 = 13 controls for N2 (Mean age = 65.46 ± 12.83 y)	High PTH and normal albumin-corrected calcium No secondary causes 25OHD > 30 ng/mL (75 nmol/L)	Osteoporosis prevalence

**Table 2:** Mineral metabolism panel amid included studies in NPHPT [35,36,37,38,39,40,41,42,43,44,45,46,47,48]; (Abbreviations: BMD = bone mineral density; Ca = calcium; Ca/Cr = calcium-to-creatinine ratio; F = female; hPHPT = hypercalcemic primary hyperparathyroidism; IQR = interquartile interval; M = male; N = number of patients; nPHPT = normocalcemic primary hyperparathyroidism; nhPHPT = normal hormonal PHPT; NA = not available; PTH = parathormone; PHPT = primary hyperparathyroidism; SD = standard deviation; vs. = versus).

Reference Number	Studied Population	Serum Total Calcium Mean ± SD or Median (IQR)	Ionized Serum Calcium Mean ± SD	Serum Phosphorus Mean ± SD or Median (IQR)	PTH Mean ± SD or Median (IQR)	24 h Urinary Calcium Mean ± SD or Median (IQR)
[35]	N = 91 with PHPT and kidney stones and hypercalciuria N1 = 56 with hPHPT N2 = 35 with nPHPT	Total Ca (mmol/L): N: 2.58 (2.47–2.67) N1: 2.63 (2.57–2.7) N2: 2.46 (2.37–2.51) ***p* < 0.0001**	N: 1.33 (1.29–1.41) mmol/L N1: 1.38 (1.35–1.43) mmol/L N2: 1.28 (1.25–1.29) mmol/L ***p* < 0.0001**	N: 0.78 (0.67–0.85) mmol/L N1: 0.77 (0.65–0.85) mmol/L N2: 0.8 (0.69–0.85) mmol/L) *p* = 0.53	N: 80 (58–109) ng/mL N1: 82 (61–113) ng/mL N2: 66 (55–94) ng/mL *p* = 0.13	N: 7.2 (4.8–11.6) mmol/24 h (288.5 (192.3–464.9) mg/24 h) N1: 7.8 (4.7–12.7) mmol/24 h (312.6 (188.3–508.9 mg/24 h) N2: 7.0 (4.9–11.2) mmol/24 h (280.5–448.8 mg/24 h) *p* = 0.75
[36]	N = 316 with PHPT N1 = 266 with hPHPT N2 = 48 with nPHPT	Albumin-adjusted Ca (mmol/L): N1: 2.85 (2.72–2.98) N2: 2.57 (2.51–2.60) ***p* < 0.001**	NA	N1: 0.89 (0.81–1.01) mmol/L N2: 0.98 ± 0.2 mmol/L ***p* = 0.006**	N1: 12.4 (8.9–20.6) pmol/L (116.93 (83.9–194.25) pg/mL) N2: 9.6 (8.3–12.8) pmol/L (90.5 (78.26–120.7) pg/mL) ***p* = 0.001**	N1: 5.8 (3.6–8.3) mmol/24 h (232.3 (144.2–332.6 mg/24 h) N2: 5.2 ± 2.4 mmol/24 h (208.4 ± 96.18 mg/24 h ***p* < 0.001**
[37]	N = 421 with PHPT referred for parathyroidectomy N1 = 340 with hPHPT N2 = 39 with nPHPT N3 = 42 with nhPHPT	Ca <11.2 mg/dL (2.79 mmol/L) N: 80.5% N1: 78.5% N2: 100% N3: 78.6% *p* = 0.99	NA	NA	NA	≤400 mg/24 h N: 82.4% N1: 83.1% N2: 89.5% N3: 70.3% *p* = 0.8
[38]	N = 105 referred for primary hyperparathyroidism, osteoporosis, or elevated PTH level with normal serum calcium N1 = 30 with hPHPT N2 = 30 with nPHPT N3 = 45 with osteoporosis without PHPT	Albumin-adjusted Ca: N: 9.9 ± 0.8 mg/dL (2.47 ± 0.2 mmol/L) N1: 10.9 ± 0.5 mg/dL (2.72 ± 0.12 mmol/L) N2: 9.5 ± 0.4mg/dL (2.37 ± 0.1 mmol/L) N3: 9.4 ± 0.3 mg/dL (2.34 ± 0.07 mmol/L) ***p* = 0.00**	NA	N: 3.5 ± 0.6 mg/dL (1.13 ± 0.19 mmol/L) N1: 2.9 ± 0.6 mg/dL (0.94 ± 0.19 mmol/L) N2: 3.4 ± 0.4 mg/dL (1.1 ± 0.13 mmol/L) N3: 3.8 ± 0.5 mg/dL (1.23 ± 0.16 mmol/L) ***p* = 0.00**	N: 107 ± 89 pg/mL N1: 164 ± 131 pg/mL N2: 129 ± 55 pg/mL N3: 55 ± 16 pg/mL ***p* = 0.000**	Urinary Ca/Cr ratio N: 0.13 ± 0.18 N1: 0.25 ± 0.19 N2: 0.14 ± 0.1 N3: 0.15 ± 0.08 ***p* = 0.01**
[39]	N = 109 with PHPT and osteoporosis who underwent parathyroidectomy N1 = 32 with hPHPT N2 = 39 with nPHPT with elevated ionized calcium N3 = 38 with nPHPT with normal ionized calcium	Total Ca (mmol/L) N: 2.51 (2.47–2.55) N1: 2.70 (2.67–2.75) N2: 2.51 (2.48–2.54) N3: 2.39 (2.37–2.42) ***p* < 0.001**	N: 1.34 (1.31–1.36) mmol/L N1: 1.43 (1.40–1.46) mmol/L N2: 1.34 (1.33–1.35) mmol/L N3: 1.28 (1.27–1.29) mmol/L ***p* < 0.001**	N: 0.93 (0.88–0.97) mmol/L N1: 0.88 (0.81–0.97) mmol/L N2: 0.89 (0.86–1.00) mmol/L N3: 0.97 (0.92–1.04) mmol/L ***p* = 0.009**	N: 8.15 (35.65–41.25) pg/mL N1: 44.15 (36.25–48.60) pg/mL N2: 39.2 (35.9–41.5) pg/mL N3: 34.9 (33.5–37.8) pg/mL *p* = 0.07 normal = (9–29) pg/mL	N: 4.15 (3.6–4.8) mmol/24 h (166.32 (144.2–192.3 mg/24 h) N1: 5.75 (4.60–6.54) mmol/24 h (230.4 (184.3–262.11) mg/24 h) N2: 4.2 (3.40–4.95) mmol/24 h (168.3 (136.2–198.3) mg/24 h) N3: 3.6 (2.5–3.9) mmol/24 h (144.2 (100.2–156.3) mg/24 h) ***p* < 0.001**
[40]	N = 170 with PHPT and controls N1 = 50 with hPHPT N2 = 40 with nPHPT N3 = 80 age-matched controls	Albumin-adjusted Ca: N: 9. 8 ± 0.7 N1: 10.8 ± 0.4 mg/dL (2.69 ± 0.1 mmol/L) N2: 9.4 ± 0.5 mg/dL (2.35 ± 0.12 mmol/L) N3: 9.4 ± 0.4 mg/dL (2.35 ± 0.1 mmol/L) ***p* < 0.001**	N: 1.3 (1.2–1.3) mmol/L N1: 1.3 (1.3–1.4) mmol/L N2: 1.2 (1.2–1.2) mmol/L N3: 1.2 (1.2–1.3) mmol/L ***p* < 0.001**	N: 3.2 ± 0.6 mg/dL (1.03 ± 0.19 mmol/L) N1: 2.8 ± 0.5 mg/dL (0.9 ± 0.16 mmol/L) N2: 3.1 ± 0.5 mg/dL (1 ± 0.16 mmol/L) N3: 3.6 ± 0.5 mg/dL (1.16 ± 0.16 mmol/L) ***p* < 0.001**	N: 81.6 (56.0–120.0) pg/mL N1: 116.9(104–153.2) pg/mL N2: 120.0 (109.3–141.0) pg/mL N3: 55.0 (44.5–64.0) pg/mL ***p* < 0.001**	NA
[41]	N = 280 (With indication for parathyroidectomy) N1 = 158 with hPHPT Mean age = 59.3 ± 14.0 y N2 = 122 with nPHPT N3 = 95 with elevated ionized Ca nPHPT N4 = 27 with normal ionized Ca nPHPT	Albumin-adjusted Ca: N1: 11.4 ± 1.1 mg/dL (2.84 ± 0.27 mmol/L) N2: 9.9 ± 0.5 mg/dL (2.47 ± 0.12 mmol/L) ***p* = 0.000** N3: 10.1 ± 0.3 mg/dL (2.52 ± 0.07 mmol/L) N4: 9.5 ± 0.6 mg/dL (2.37 ± 0.15 mmol/L) ***p* = 0.000** N1 vs. N4: ***p* < 0.001**	N1: 5.8 ± 0.4 mg/dL (1.45 ± 0.1 mmol/L) N2: 5.4 ± 0.3 mg/dL (1.35 ± 0.07 mmol/L) ***p* = 0.000** N3: 5.5 ± 0.2 mg/dL (1.37 ± 0.05 mmol/L) N4: 4.9 ± 0.3 mg/dL (1.22 ± 0.07 mmol/L) ***p* = 0.000** N1 vs. N4: ***p* < 0.001**	N1: 2.6 ± 0.5 mg/dL (0.84 ± 0.16 mmol/L) N2: 2.9 ± 0.5 mg/dL(0.93 ± 0.16 mmol/L) ***p* = 0.000** N3: 2.8 ± 0.5 mg/dL (0.9 ± 0.16 mmol/L) N4: 3.2 ± 0.5 mg/dL (1.03 ± 0.16 mmol/L) ***p* = 0.000** N1 vs. N4: ***p* < 0.001**	N1: 212.7 ± 219.3 pg/mL N2: 115.9 ± 45.7 pg/mL ***p* = 0.000** N3: 120.6 ± 49.2 pg/mL N4: 99.1 ± 24.5 pg/mL ***p* = 0.002** N1 vs. N4: ***p* = 0.008**	N1: 324.3 ± 178.2 mg/24 h N2: 285.8 ± 159.9 mg/24 h *p* = 0.105 N4: 315.7 ± 169.3 mg/24 h N1 vs. N4: ***p* = 0.848**
[42]	N = 87 with PHPT referred for parathyroidectomy (at the indication of an endocrinologist) (30 lost to follow-up) N1 = 71 with hPHPT (28 lost to follow-up) N2 = 16 with nPHPT (2 lost to follow-up)	Albumin-adjusted Ca: N1: 10.8 ± 0.7 mg/dL (2.69 ± 0.17 mmol/L) N2: 9.8 ± 0.3 mg/dL (2.45 ± 0.07 mmol/L) ***p* = 0.001**	N1: 5.6 ± 0.4 mg/dL (1.4 ± 0.1 mmol/L) N2: 4.9 ± 0.5 mg/dL (1.22 ± 0.12 mmol/L) ***p* = 0.001**	NA	N1: 209.7 ± 127.1 N2: 153.3 ± 28.9 *p* = 0.07	N1: 286.6 ± 104.6 mg/24 h N2: 314 ± 116.6 mg/24 h *p* = 0.55
[43]	N = 104 with PHPT who underwent parathyroidectomy N1 = 88 with hPHPT N2 = 16 with nPHPT	Albumin-adjusted Ca (mmol/L): N1: 2.7 ± 0.14 N2: 2.41 ± 0.06 ***p* < 0.001**	N1: 1.39 ± 0.09 mmol/L N2: 1.19 ± 0.02 mmol/L ***p* < 0.001**	NA	N1: 20.8 ± 11.2 pmol/L (196.14 ± 105.2 pg/mL) N2: 15.9 ± 4.1 pmol/L (149.9 ± 38.6 pg/mL) *p* = 0.06	N1: 294.4 ± 110 mg/24 h N2: 313.7 ± 122.4 mg/24 h *p* = 0.53
[44]	N = 750 men (population sample) N1 = 3 with hPHPT N2 = 21 with nPHPT N3 = 3 with secondary HPT N4 = 680 with normal PTH N5 = 312 with normal calcium, PTH and vitamin D	Albumin-adjusted Ca (mmol/L): N2: 2.26 ± 0.07 N5: 2.30 ± 0.07 ***p* = 0.025**	NA	NA	N2: 8.04 ± 0.88 pmol/L (75.8 ± 8.3 pg/mL) N5: 4.41 ± 0.97 pmol/L (41.58 ± 9.14 pg/mL) ***p* < 0.001**	NA
[45]	N = 43 women with PHPT N1 = 29 with hPHPT N2 = 7 with nPHPT N3 = 7 controls	Albumin-adjusted Ca: N1: 10.5 ± 0.5 mg/dL (2.62 ± 0.12) mmol/L) N2: 9.4 ± 0.9 mg/dL (2.35 ± 0.22 mmol/L) N3: 9.5 ± 0.9 mg/dL (2.37 ± 0.22 mmol/L) ***p* < 0.001**	NA	N1: 3.2 ± 0.5 mg/dL (1.03 ± 0.16 mmol/L) N2:3.3 ± 1.1 mg/dL (1.06 ± 0.35 mmol/L) N3:3.7 ± 1.1 mg/dL (1.19 ± 0.35 mmol/L) *p* = 0.11	N1: 93 ± 6 pg/mL N2: 102 ± 121 pg/mL N3: 36 ± 11 pg/mL ***p* = 0.002**	NA
[46]	N = 127 with PHPT and controls N1 = 41 with hPHPT N2 = 47 with nPHPT N3 = 39 controls	Albumin-adjusted Ca: N1: 10.8 ± 0.4 mg/dL (2.69 ± 0.1 mmol/L) N2: 9.4 ± 0.4 mg/dL (2.35 ± 0.1 mmol/L) N3: 9.5 ± 0.4 mg/dL (2.37 ± 0.1 mmol/L) N1 vs. N3: ***p* < 0.001** N1 vs. N2: ***p* < 0.001**	N1: 1.35 ± 0.05 mmol/L N2: 1.19 ± 0.05 mmol/L N3: 1.22 ± 0.05 mmol/L N1 vs. N3: ***p* < 0.001** N2 vs. N3: ***p* < 0.05** N1 vs. N2: ***p* < 0.001**	N1: 2.8 ± 0.5 mg/dL (0.9 ± 0.16 mmol/L) N2: 3.2 ± 0.5 mg/dL (1.03 ± 0.16 mmol/L) N3: 3.8 ± 0.4 mg/dL (1.22 ± 0.13 mmol/L) N1 vs. N3: *p* < 0.001 N2 vs. N1: ***p* < 0.001** N2 vs. N3: ***p* < 0.001**	N1: 139.1 ± 49.7 pg/mL N2: 126.8 ± 29.5 pg/mL N3: 52.4 ± 15.4 pg/mL N1 vs. N3: ***p* < 0.001** N2 vs. N3: ***p* < 0.001**	N1: 293.5 ± 146.3 mg/24 h N2: 196.1 ± 49.2 mg/24 h N3: 192.3 ± 76 mg/24 h N1 vs. N3: ***p* < 0.001** N1 vs. N2: ***p* < 0.001**
[47]	N = 6280 referred for BMD measurements N1 = 17 with hPHPT N2 = 11 with nPHPT N3 = 300 controls F:M = 214:86	Albumin-adjusted Ca (mmol/L): N1: 2.75 ± 0.11 N2: 2.55 ± 0.05 N3: 2.37 ± 0.08 ***p* < 0.001**	NA	N1: 0.89 ± 0.16 mmol/L N2: 1.04 ± 0.14 mmol/L N3: 1.12 ± 0.18 mmol/L ***p* < 0.001**	N1: 102.4 (89.0, 112.4) pg/mL N2: 106.8 (86.9, 123.9) pg/mL N3: 42.5 (40.8, 44.2) pg/mL ***p* < 0.001**	NA
[48]	N = 40 postmenopausal women with PHPT and controls N1 = 7 with hPHPT N2 = 13 with nPHPT N3 = 7 controls for N1 N4 = 13 controls for N2	Albumin-adjusted Ca: N1: 10.91 ± 0.38 mg/dL (2.72 ± 0.09 mmol/L) N3: 9.03 ± 0.32 mg/dL (2.25 ± 0.08 mmol/L) ***p* < 0.001** N2: 9.42 ± 0.48 mg/dL (2.35 ± 0.12 mmol/L) N4: 9.13 ± 0.48 mg/dL (2.28 ± 0.12 mmol/L) *p* = 0.181 N1 vs. N2: ***p* = 0.001**	NA	N1: 3.31 ± 0.93 mg/dL (1.06 ± 0.3 1.11 ± 0.1 mmol/L) N3: 3.46 ± 0.83 mg/dL (1.11 ± 0.26 mmol/L) *p* = 0.782 N2: 3.55 ± 0.41 mg/dL (1.14 ± 0.13 mmol/L) N4: 3.52 ± 0.42 mg/dL (1.13 ± 0.13 mmol/L) *p* = 0.874 N1 vs. N2: *p* = 0.267	N1: 127.87 ± 64.88 pg/mL N3: 55.81 ± 12.53 pg/mL ***p* = 0.039** N2: 84.75 ± 13.37 pg/mL N4: 45.48 ± 11.12 pg/mL ***p* = 0.001** N1 vs. N2: *p* = 0.190	N1: 248.14 ± 179.32 mg/24 h N3: 177.31 ± 71.03 mg/24 h *p* = 0.499 N2: 136.31 ± 90.66 mg/24 h N4: 110.13 ± 61.65 mg/24 h *p* = 0.399 N1 vs. N2: *p* = 0.052

**Table 3:** Vitamin D findings in patients diagnosed with NPHPT via study-based analysis according to our methods [35,36,37,39,40,41,42,43,44,45,46,47,48] (Abbreviations: 25OHD = 25-hydroxyvitamin D; Ca = calcium; hPHPT = hypercalcemic primary hyperparathyroidism; IQR = interquartile range; nPHPT = normocalcemic primary hyperparathyroidism; N = number of patients; NA = not available; PHPT = primary hyperparathyroidism; PTH = parathormone; SD = standard deviation; vs. = versus).

Reference Number	Studied Population	25OHD Threshold for Sufficiency	Vitamin D Status (25-Hydroxyvitamin D Assays)	
			Mean ± SD or Median (IQR)	*p*-Value Between the Analyzed Subgroups
[35]	N = 91 with PHPT and kidney stones and hypercalciuria N1 = 56 with hPHPT N2 = 35 with nPHPT	NA	N: 24 (17–30) ng/mL N1: 21 (12–25) ng/mL N2: 26 (23–37) ng/mL	***p* = 0.04**
[36]	N = 316 with PHPT N1 = 266 with hPHPT N2 = 48 with nPHPT	>20 ng/mL	N1: 20.1 (13.1–26.6) ng/mL N2: 25.9 (22.3–31.3) ng/mL	***p* < 0.001**
[37]	N = 105 patients with PHPT (surgery candidates) N1 = 30 with hPHPT N2 = 30 with nPHPT N3 = 45 with osteoporosis without PHPT	>60 nmol/L (24 ng/mL)	N: 87 ± 29 nmol/L (24.86 ± 11.62 ng/mL) N1: 89 ± 37 nmol/L (35.66 ± 14.82 ng/mL) N2: 85 ± 19 nmol/L (34 ± 7.61 ng/mL) N3: 86 ± 28 nmol/L (34.46 ± 11.22 ng/mL)	*p* = 0.88
[39]	N = 109 with PHPT and osteoporosis who underwent parathyroidectomy N1 = 32 with hPHPT N2 = 39 with nPHPT with elevated ionized calcium N3 = 38 with nPHPT with normal ionized calcium	>30 ng/mL	N: 37.2 (34–40) ng/mL N1: 31 (27–34.8) ng/mL N2: 35.5 (32.25–41.0) ng/mL N3: 40 (39.5–43.2) ng/mL	***p* < 0.001**
[40]	N = 170 with PHPT and controls N1 = 50 with hPHPT N2 = 40 with nPHPT N3 = 80 age-matched controls	>30 ng/mL	N: 33.0 (27.0–38.0) ng/mL N1: 32.7 (27.0–36.4) ng/mL N2: 35.3 (32.0–37.9) ng/mL (Oral cholecalciferol supplementation in N2) N3: 31.0 (23.5–40.0) ng/mL	***p* = 0.013**
[41]	N = 280 with PHPT (surgery candidates) N1 = 158 with hPHPT N2 = 122 with nPHPT N3 = 95 with elevated ionized Ca nPHPT N4 = 27 with normal ionized Ca nPHPT	NA	N1: 19.3 ± 9.4 ng/mL N2: 21.4 ± 9.2 ng/mL N4: 20.1 ± 8.6 ng/mL	N1 vs. N2: *p* = 0.065 N1 vs. N4: *p* = 0.682
[42]	N = 87 with PHPT (30 lost to follow-up) N1 = 71 with hPHPT (28 lost to follow-up) N2 = 16 with nPHPT (2 lost to follow-up)	>30 ng/mL	N1: 21.2 ± 10.2 nmol/L (8.49 ± 4.08 ng/mL) N2: 32 ± 5.8 nmol/L (12.82 ± 2.32 ng/mL)	***p* = 0.001**
[43]	N = 104 with PHPT who underwent parathyroidectomy N1 = 88 with hPHPT N2 = 16 with nPHPT	>30 ng/mL	N1: 21.5 ± 11.1 nmol/L (8.61 ± 4.44 ng/mL) N2: 33.1 ± 5.7 nmol/L (13.26 ± 2.28 ng/mL)	***p* < 0.001**
[44]	N = 750 men with PHPT N1 = 3 with hPHPT N2 = 21 with nPHPT N3 = 3 with secondary HPT N4 = 680 with normal PTH N5 = 312 with normal calcium, PTH and vitamin D	≥50 nmol/L (20 ng/mL)	N2: 63.1 ± 10.4 nmol/L (25.28 ± 4.166 ng/mL) N5: 64.4 ± 14.2 nmol/L (25.8 ± 5.68 ng/mL)	*p* = 0.596
[45]	N = 43 women with PHPT N1 = 29 with hPHPT N2 = 7 with nPHPT N3 = 7 controls	≥20 ng/mL	N1: 37.1 ± 15.4 ng/mL N2: 38.1 ± 30.7 ng/mL N3: 36.6 ± 30.7 ng/mL	*p* = 0.82
[46]	N = 127 with PHPT N1 = 41 with hPHPT N2 = 47 with nPHPT N3 = 39 controls	>30 ng/mL	N1: 31.1 ± 7.8 ng/mL N2: 36.7 ± 6.6 ng/mL N3: 28.6 ± 12.8 ng/mL	N1 vs. N3: ***p* < 0.05** N2 vs. N3: ***p* < 0.001**
[47]	N = 6280, including: N1 = 17 with hPHPT N2 = 11 with nPHPT N3 = 300 controls	≥50 nmol/L (20 ng/mL)	N1: 71.4 (30.5) nmol/L (28.6 (12.22) ng/mL) N2: 62.8 (23.5) nmol/L (25.16 (9.4) ng/mL) N3: 78.9 (32.9) nmol/L (31.6 (13.18) ng/mL)	*p* = 0.83
[48]	N = 40 postmenopausal women with PHPT N1 = 7 with hPHPT N2 = 13 with nPHPT N3 = 7 controls for N1 N4 = 13 controls for N2	>30 ng/mL	N1: 35.07 ± 5.57 ng/mL N3: 35.50 ± 10.58 ng/mL N2: 39.03 ± 7.87 ng/mL N4: 36.92 ± 12.98 ng/mL	N1 vs. N3: *p* = 0.942 N2 vs. N4: *p* = 0.5

**Table 4:** Prevalence of osteoporosis/osteopenia in patients with NPHPT at baseline [36,37,39,41,45,48] (Abbreviations: BMD = bone mineral density; DXA = Dual-Energy X-Ray Absorptiometry; hPHPT = hypercalcemic primary hyperparathyroidism; N = number of patients; NA = not available; nPHPT = normocalcemic primary hyperparathyroidism; nhPHPT = normal hormonal primary hyperparathyroidism; PHPT = primary hyperparathyroidism).

Reference Number	Studied Population	Osteoporosis Prevalence	Osteopenia Prevalence	Osteoporosis Criteria
[36]	N = 316 with PHPT N1 = 266 with hPHPT N2 = 48 with nPHPT	N1: 44.7% N2: 41.7% *p* = 0.575		NA
[37]	N = 421 with PHPT referred for parathyroidectomy N1 = 340 with hPHPT N2 = 39 with nPHPT N3 = 42 with nhPHPT	N1: 37.7% N2: 58.8% N3: 38.2%	N1:51.4% N2:32.4% N3:47.1%	DXA T-score
[39]	N = 109 with PHPT and osteoporosis who underwent parathyroidectomy N1 = 32 with hPHPT N2 = 39 with nPHPT with elevated ionized calcium N3 = 38 with nPHPT with normal ionized calcium	Osteoporosis was an inclusion criterion (defined by osteoporotic fracture and/or T-score ≤ −2.5) Criteria based on T-score ≤ −2.5: N1: 69% N2: 77% N3: 97% ***p* = 0.03**		Osteoporotic fracture and/or a T-score below −2.5SD
[41]	N = 280 (with indication for parathyroidectomy) N1 = 158 with hPHPT N2 = 122 with nPHPT N3 = 95 with elevated ionized Ca nPHPT N4 = 27 with normal ionized Ca nPHPT	N1: 50% N2: 42.4% N3: 38.8% N4: 57.9% N1 vs. N2: ***p* = 0.008** N3 vs. N4: *p* = 0.074 N1 vs. N4: ***p* = 0.012**	N1: 38.6% N2: 30.3% N3: 35% N4: 10.5%	DXA-BMD
[45]	N = 43 women with PHPT N1 = 29 with hPHPT N2 = 7 with nPHPT N3 = 7 controls	N1: 31% N2: 100% ***p* = 0.008**		NA
[48]	N = 40 postmenopausal women with PHPT and controls N1 = 7 with hPHPT N2 = 13 with nPHPT N3 = 7 controls for N1 N4 = 13 controls for N2	N1: 57.1% N2: 53.8% N3: 0% N4: 23.1% N2 vs. N4: *p* = 0.072	N1: 28.6% N2: 38.5% N3: 42.9% N4: 69.2%	DXA-BMD

**Table 5:** DXA-based findings in patients with NPHPT [36,38,40,41,42,43,44,45,46,47] (Abbreviations: BMD = bone mineral density; F = female; hPHPT = hypercalcemic primary hyperparathyroidism; M = male; NA = not available; nPHPT = normocalcemic primary hyperparathyroidism; nhPHPT = normal hormonal primary hyperparathyroidism; N = number of patients; y = years; vs. = versus).

Reference Number	Studied Population	Lumbar BMD and T-Score (or Z-Score)	Femoral Neck/Hip BMD and T-Score (or Z-Score)	1/3 Radius BMD and T-Score
[36]	N1 = 266 with hPHPT F:M = 234:32 Mean age = 59.0 ± 11.8 y N2 = 48 with nPHPT F:M = 42:6 Mean age = 56.9 ± 13.4 y	BMD (g/cm^2^) N1: 0.91 (0.80–1.00) N2: 0.92 (0.82–1.06) *p* = 0.690	Femoral neck BMD (g/cm^2^) N1: 0.76 (0.65–0.86) N2: 0.78 (0.68–0.86) *p* = 0.530 Total hip BMD (g/cm^2^) N1: 0.77 (0.71–0.91) N2: 0.82 (0.75–0.97) *p* = 0.533	BMD (g/cm^2^) N1: 0.51 (0.44–0.58) N2: 0.54 (0.48–0.58) *p* = 0.957
[38]	N1 = 30 with hPHPT F:M = 27:3 Mean age = 69.3 ± 9.1 y N2 = 30 with nPHPT F:M = 28:2 Mean age = 69.7 ± 7.2 y N3 = 45 with osteoporosis without PHPT F:M = 43:2 Mean age = 68.4 ± 7.7 y	BMD (g/cm^2^) N1: −0.99 ± 0.16 N2: −0.97 ± 0.13 N3: −0.99 ± 0.16 *p* = 0.13 T-score N1: −1.7 ± 1 N2: −1.9 ± 0.9 N3: −2.3 ± 0.9 ***p* = 0.04**	Femoral neck BMD (g/cm^2^) BMD (g/cm^2^) N1: 0.795 ± 0.14 N2: 0.777 ± 0.1 N3: 0.75 ± 0.088 *p* = 0.313 Femoral neck T-score N1: −2.2 ± 0.88 N2: −2.1 ± 0.66 N3: −2.2 ± 0.49 *p* = 0.6	NA
[40]	N1 = 50 with hPHPT F:M = 47:3 Mean age = 65.2 ± 11.6 y N2 = 40 with nPHPT F:M = 37:3 Mean age = 63.4 ± 9.0 y N3 = 80 age-matched controls F:M = 75:5 Mean age = 65.4 ± 7.8 y	BMD (g/cm^2^) N1: 0.82 ± 0.19 N2: 0.88 ± 0.18 N3: 0.87 ± 0.14 *p* = 0.327	Femoral neck BMD (g/cm^2^) N1: 0.63 ± 0.11 N2: 0.67 ± 0.11 N3: 0.67 ± 0.08 *p* = 0.101 Total hip BMD (g/cm^2^) N1: 0.79 ± 0.14 N2: 0.84 ± 0.12 N3: 0.84 ± 0.10 *p* = 0.1	NA
[41]	N1 = 158 with hPHPT F:M = 120:38 Mean age = 59.3 ± 14.0 y N2 = 122 with nPHPT F:M = 105:17 Mean age = 54.3 ± 13.1 y	T-score N1: −2.4 ± 1.2 N2: −2.0 ± 1.3 ***p* = 0.024**	NA	NA
[42]	N1 = 71 with hPHPT (28 lost to follow-up) F:M = 55:16 Mean age = 61.4 ± 11 y N2 = 16 with nPHPT (2 lost to follow-up) F:M = 13:3 Mean age = 61.6 ± 11 y	BMD (g/cm^2^) N1: 0.8 ± 0.1 N2: 0.8 ± 0.2 *p* = 0.7 T-score N1: −2.1 ± 1.3 N2: −2.3 ± 1.5 *p* = 0.62	Femoral neck BMD (g/cm^2^) N1: 0.7 ± 0.1 N2: 0.6 ± 0.1 *p* = 0.08 Femoral neck T-score N1: −1.7 ± 0.9 N2: −1.9 ± 1.1 *p* = 0.36	BMD (g/cm^2^) N1: 0.5 ± 0.1 N2: 0.5 ± 0.1 *p* = 0.7 T-score N1: −2.0 ± 1.3 N2: −2.2 ± 1.2 *p* = 0.7
[43]	N1 = 88 with hPHPT F:M = 68:20 Mean age = 60.6 ± 11 y N2 = 16 with nPHPT F:M = 13:3 Mean age = 60.9 ± 10.4 y	T-score N1: 2.1 ± 1.1 N2: 2.4 ± 0.9 *p* = 0.4	Femoral neck T-score N1: 1.8 ± 1.0 N2: 2.0 ± 0.9 *p* = 0.3	T-score N1: 2.2 ± 1.2 N2: 2.0 ± 0.9 *p* = 0.95
[46]	N1 = 41 with hPHPT F:M = 38:3 Mean age = 63.9 ± 12 y N2 = 47 with nPHPT F:M = 43:4 Mean age = 63.8 ± 9.3 y N3 = 39 controls F:M = 35:4 Mean age = 64.7 ± 7 y	BMD (g/cm^2^) N1: 0.880 ± 0.184 N2: 0.893 ± 0.186 N3: 0.904 ± 0.149 *p* > 0.05 (N2 vs. N1, N2 vs. N3, N3 vs. N1) T-score N1: −1.5 ± 1.6 N2: −1.4 ± 1.7 N3: −1.3 ± 1.3 *p* > 0.05 (N2 vs. N1, N2 vs. N3, N3 vs. N1)	Femoral neck BMD (g/cm^2^) N1: 0.633 ± 0.107 N2: 0.659 ± 0.108 N3: 0.671 ± 0.075 *p* > 0.05 (N2 vs. N1, N2 vs. N3, N3 vs. N1) Femoral neck T-score N1: −2.0 ± 1.0 N2: −1.8 ± 0.9 N3: −1.6 ± 0.7 *p* > 0.05 (N2 vs. N1, N2 vs. N3, N3 vs. N1) Total hip BMD (g/cm^2^) N1: 0.795 ± 0.126 N2: 0.819 ± 0.125 N3: 0.872 ± 0.097 ***p* < 0.05** (N1 vs. N3) Total hip T-score N1: −1.2 ± 1 N2: −1.1 ± 0.9 N3: −0.6 ± 0.7 ***p* < 0.05** (N1 vs. N3)	BMD (g/cm^2^) N1: 0.563 ± 0.078 N2: 0.605 ± 0.08 N3: 0.620 ± 0.065 ***p* < 0.05** (N1 vs. N2, N1 vs. N3) T-score N1: −2.3 ± 1.3 N2: −1.6 ± 1.2 N3: −1.3 ± 0.8 ***p* < 0.05** (N1 vs. N2) ***p* < 0.001** (N1 vs. N3)
[47]	N1 = 17 with hPHPT F:M = 15:2 Mean age = 67 ± 6 y N2 = 11 with nPHPT F:M = 10:1 Mean age = 68 ± 11 y N3 = 300 controls F:M = 214:86 Mean age = 70 ± 20 y	Z-score N1: −0.2 ± 1.3 N2: 0.2 ± 2.2 N3: −0.1 ± 1.7 *p* = 0.932	Femoral neck Z-score N1: −0.4 ± 0.8 N2: −0.1 ± 1.3 N3: −0.4 ± 1.0 *p* = 0.770	NA

**Table 6:** Trabecular bone score and bone strain index across our search [46]. (Abbreviations: BMD = bone mineral density; F = female, hPHPT = hypercalcemic primary hyperparathyroidism; nPHPT = normocalcemic primary hyperparathyroidism; N = number of patients; M = male; TBS = trabecular bone score; y = years; vs. = versus).

Reference Number	Studied Population	TBS	Bone Strain Index: Lumbar Spine	Bone Strain Index: Femoral Neck	Bone Strain Index: Total Hip
[46]	N1 = 50 with hPHPT F:M = 47:3 (Mean age = 65.2 ± 11.6 y) N2 = 40 with nPHPT F:M = 37:3 (Mean age = 63.4 ± 9.0 y) N3 = 80 age-matched controls F:M = 75:5 (Mean age = 65.4 ± 7.8 y)	N1: 1.24 ± 0.10 N2: 1.29 ± 0.14 N3: 1.30 ± 0.07 N1 vs. N2: *p* > 0.05 N1 vs. N3: ***p* = 0.009** N2 vs. N3: *p* > 0.05	BMD (g/cm^2^) N1: 2.28 ± 0.60 N2: 2.11 ± 0.65 N3: 2.01 ± 0.44 ***p* = 0.023** N1 vs. N2: *p* > 0.05 N1 vs. N3: ***p* = 0.017** N2 vs. N3: *p* > 0.05	BMD (g/cm^2^) N1: 1.72 ± 0.42 N2: 1.52 ± 0.31 N3: 1.47 ± 0.35 ***p* = 0.001** N1 vs. N2: ***p* = 0.031** N1 vs. N3: ***p* = 0.001** N2 vs. N3: *p* > 0.05	BMD (g/cm^2^) N1: 1.52 ± 0.34 N2: 1.36 ± 0.23 N3: 1.34 ± 0.26 ***p* = 0.001** N1 vs. N2: ***p* = 0.030** N1 vs. N3: ***p* = 0.001** N2 vs. N3: *p* > 0.05

**Table 7:** Prevalent fractures and fracture risk assessment in NPHPT [36,37,38,39,40,41,44,45,46]. (Abbreviations: hPHPT = hypercalcemic primary hyperparathyroidism; nPHPT = normocalcemic primary hyperparathyroidism; nhPHPT = normal hormonal primary hyperparathyroidism; N = number of patients; OR = odds ratio; RR = risk ratio; y = years; vs. = versus).

Reference Number	Studied Subgroups	History of Osteoporotic Fracture as Defined by the Original Authors
[36]	N1 = 266 with hPHPT N2 = 48 with nPHPT	Low energy fractures: N1: 7% vs. N2: 8.3% (*p* = 0.483)
[37]	N1 = 340 with hPHPT N2 = 39 with nPHPT N3 = 42 with nhPHPT	Previous fractures: N1: 9.8% vs. N2: 12.8% vs. N3: 24.4% (***p* = 0.02**)
[38]	N1 = 30 with hPHPT N2 = 30 with nPHPT N3 = 45 with osteoporosis without PHPT	Previous fractures: N1: 6.7% vs. N2: 26.7% vs. N3: 40% (*p* = 0.06)
[39]	N1 = 32 with hPHPT N2 = 39 with nPHPT with elevated ionized calcium N3 = 38 with nPHPT with normal ionized calcium	Fracture in the past 5 y: N1:34% vs. N2:41% vs. N3: 42% (*p* = 0.3)
[40]	N1 = 50 with hPHPT N2 = 40 with nPHPT N3 = 80 age-matched controls	Vertebral fractures: N1: 36.7% vs. N2: 20% vs. N3: 12.5% (N1 vs. N2: *p* = 0.005; N1 vs. N3: ***p* < 0.05**) Moderate–severe vertebral fractures: N1: 20.4% vs. N2: 5.0% vs. N3: 5.1% (N1 vs. N2: ***p* < 0.05**; N1 vs. N3: ***p* < 0.05**)
[41]	N1 = 158 with hPHPT N2 = 122 with nPHPT N3 = 95 with elevated ionized Ca nPHPT N4 = 27 with normal ionized Ca nPHPT	Prevalent fractures: N1: 8.2% vs. N2: 7.4% vs. N3: 7.4% vs. N4: 7.4% (N1 vs. N2: *p* = 0.793; N3 vs. N4: *p* = 0.995; N1 vs. N4: *p* = 0.885)
[44]	N1 = 3 with hPHPT N2 = 21 with nPHPT N3 = 3 with secondary HPT N4 = 680 with normal PTH N5 = 312 with normal calcium, PTH and vitamin D	After 21 years (any fracture): N2: 5% vs. N5: 6% (*p* > 0.5)
[45]	N1 = 29 with hPHPT N2 = 7 with nPHPT	Fragility fractures: N1: 13.8% vs. N2: 42.8% (*p* = 0.23)
[46]	N1 = 41 with hPHPT N2 = 47 with nPHPT N3 = 39 controls	Vertebral fractures: N1: 60% vs. N2: 28% vs. N3: 23% (N3 vs. N1: ***p* < 0.05**) Fracture risk (vs. N3) N1: OR = 5.87 (2.16–17.3) vs. N2: OR = 1.32 (0.48–3.72) Moderate–severe fracture risk (vs. N3) N1: OR = 3.81 (1.15–15.12) vs. N2: OR = 1.04 (0.25–4.55) >1 fractures risk (vs. N3) N1: RR = 2.24 (1.22–4.32) vs. N2: RR = 1.12 (0.56–2.27) >1 moderate–severe fractures risk (vs. N3) N1: RR = 2.60 (0.99–8.08) vs. N2: RR = 0.83 (0.23–2.97)

**Table 8:** Bone turnover markers in NPHPT [35,36,38,42,43,46,47]. (Abbreviations: BALP = bone alkaline phosphatase; β-CTX = β-cross-linked telopeptide of type I collagen; hPHPT = hypercalcemic hyperparathyroidism; N = number of patients; nPHPT = normocalcemic primary hyperparathyroidism; PHPT = primary hyperparathyroidism, P1NP = procollagen type I N-terminal propeptide, vs. = versus).

Reference Number	Studied Subgroups	Bone Formation Markers			Bone Resorption Markers
		ALP or BALP * (IU/L) or ALP Activity ** mean ± SD or Median (IQR)	P1NP (ng/mL) Mean ± SD or Median (IQR)	Osteocalcin (ng/mL) Mean ± SD or Median (IQR)	β-CTX (ng/mL or pmol/L ***) Mean ± SD or Median (IQR)
[35]	N1 = 56 with hPHPT N2 = 35 with nPHPT	N1: 17 (12–24) * N2: 15 (11–23) *p* = 0.29			
[36]	N1 = 266 with hPHPT N2 = 48 with nPHPT	N1: 94.1 (74.0–116.1) N2: 86.9 ± 29.6 ***p* = 0.016**			N1: 0.84 (0.54–1.39) N2: 0.89 ± 0.5 *p* = 0.483
[38]	N1 = 30 with hPHPT N2 = 30 with nPHPT N3 = 45 with osteoporosis without PHPT		N1: 51 ± 24.6 N2: 42.5 ± 28 N3: 41 ± 20 *p* = 0.62		N1: 0.46 ± 0.33 N2: 0.3 ± 0.22 N3: 0.31 ± 0.24 *p* = 0.37
[39]	N1 = 32 with hPHPT N2 = 39 with nPHPT with elevated ionized calcium N3 = 38 with nPHPT with normal ionized calcium	N1: 69 (64–79) *** N2: 64 (58–75) N3: 60 (52–72) *p* = 0.07		N1: 34 (39.7–40.2) N2: 32 (24.6–35.8) N3: 29 (24.9–34.0) *p* = 0.1	N1: 653 (572–845) *** N2: 639 (582–745) N3: 643 (570–767) *p* = 0.9
[42]	N1 = 71 with hPHPT (28 lost to follow-up) N2 = 16 with nPHPT (2 lost to follow-up)	N1: 112.6 ± 94.4 *** N2: 91.9 ± 35.9 *p* = 0.4	N1: 71.2 ± 30.6 N2: 55.4 ± 30.2 ***p* = 0.03**	N1: 37 ± 17.4 N2: 24.4 ±11.2 ***p* = 0.007**	N1: 0.7 ± 0.4 N2: 0.4 ± 0.3 ***p* = 0.01**
[43]	N1 = 88 with hPHPT N2 = 16 with nPHPT	N1: 104.7 ± 65.5 N2: 86.1 ± 31.2 *p* = 0.1	N1: 73.5 ± 32.1 N2: 49.2 ± 24.8 ***p* = 0.005**	N1: 37.4 ± 17.1 N2: 23.5 ± 8.7 ***p* = 0.02**	N1: 0.68 ± 0.35 N2: 0.38 ± 0.2 ***p* = 0.001**
[46]	N1 = 41 with hPHPT N2 = 47 with nPHPT N3 = 39 controls		N1: 73.09 ± 42.09 N2: 61.33 ± 25.41 N3: 50.12 ± 24.14 ***p* < 0.05 (N1 vs. N3)**		N1: 0.49 ± 0.27 N2: 0.37 ± 0.18 N3: 0.33 ± 0.21 ***p* < 0.05 (N1 vs. N3)**
[47]	N1 = 17 with hPHPT N2 = 11 with nPHPT N3 = 300 controls	N1: 88 ± 27 N2: 98 ± 33 N3: 78 ± 37 *p* = 0.070			

* ALP or BLP, ** ALP Activity, *** Beta-CTX.

**Table 9:** Changes in BMD and bone turnover markers following parathyroidectomy in NPHPT [42,43]. (Abbreviations: β-CTX = β-cross-linked telopeptide of type I collagen; BMD = bone mineral density; BTM = bone turnover markers; F = females; hPHPT = hypercalcemic hyperparathyroidism; M = males; N = number of patients; nPHPT = normocalcemic primary hyperparathyroidism; PHPT = primary hyperparathyroidism; P1NP = procollagen type I N-terminal propeptide; y = years).

Reference Number	Study Design, Studied Population	DXA-BMD	Bone Turnover Markers
[42]	Prospective N = 87 with PHPT referred for parathyroidectomy (at the indication of an endocrinologist) (30 lost to follow-up) F:M = 68:19 N1 = 71 with hPHPT (28 lost to follow-up) F:M = 55:16 Mean age = 61.4 ± 11 y N2 = 16 with nPHPT (2 lost to follow-up) F:M = 13:3 Mean age = 61.6 ± 11 y	At 12 months: Lumbar spine—mean increase N1: 3.6% (0.03 g/cm^2^, [0.018 to 0.042], ***p* < 0.001**) N2: 2.8% (0.024 g/cm^2^, [−0.016 to 0.063], *p* = 0.05) Femoral neck—mean increase N1: 3.3% (0.022 g/cm^2^, [−0.004 to 0.0478], ***p* < 0.001**) N2: 4.2% (0.045 g/cm^2^, [−0.009 to 0.117], ***p* < 0.001**) Third distal radius—mean increase N1: 0.2% (−0.001 g/cm^2^; [−0.006 to 0.0035], *p* > 0.05) N2: −2.1% (−0.011 g/cm^2^; [−0.036 to 0.0144], *p* > 0.05) At 24 months: Improvement only for N1 in lumbar spine BMD: 1.1% (0.01 g/cm^2^, [−0.01 to 0.031], ***p* = 0.02**	At 12 months: N: osteocalcin: −17.7 (−21 to 14.4) P1NP: −33 (−39 to 26.5) BCTX −0.37 (−0.44 to 0.3) BTM remained in the normal range for N1 and N2
[43]	Comparative prospective N = 104 with PHPT who underwent parathyroidectomy N1 = 88 with hPHPT F:M = 68:20 Mean age = 60.6 ± 11 y N2 = 16 with nPHPT F:M = 13:3 Mean age = 60.9 ± 10.4 y	At 12 months: Lumbar spine T-score N1: 1.7 ± 1 N2: 1.6 ± 0.9 *p* = 0.92 Femoral neck T-score N1: 1.7 ± 1 N2: 1.6 ± 0.9 *p* = 0.9 Third distal radius N1: 2 ± 1.4 N2: 1.7 ± 1.25 *p* = 0.09	At 12 months: Alkaline phosphatase activity (IU/L) N1: 71.3 ± 27.5 N2: 61.6 ± 7 *p* = 0.4 Osteocalcin (ng/mL) N1: 15.8 ± 6.6 N2: 16.2 ± 4.8 *p* = 0.9 β-CTX (ng/mL) N1: 0.26 ± 0.1 N2: 0.31 ± 0.2 *p* = 0.54 P1NP (ng/mL) N1: 31.6 ± 14 N2: 35.7 ± 4.5 *p* = 0.52

## Data Availability

Not applicable.

## Supplementary Materials

The following supporting information can be downloaded at https://www.mdpi.com/article/10.3390/jcm13216325/s1. Table S1. The included studies in the final analysis according to our methods [35,36,37,38,39,40,41,42,43,44,45,46,47,48].
