# Supplementary material for: Osteoporosis and Normocalcemic Primary Hyperparathyroidism (Conservatively or Surgically Managed)
Source: J Clin Med. 2024 Oct 23;13(21):6325. doi: 10.3390/jcm13216325 (PMC11545940; doi:10.3390/jcm13216325)
Supplement: Supplementary file 1 [file jcm-13-06325-s001.zip › jcm-3257424-supplementary.pdf]

Table S1. The included studies in the final analysis according to our methods [35–48].

Study number	Reference number	First author	Year of publication	Study design	Studied sub-groups
1	[35]	Halimi	2024	Retrospective	N=91 with PHPT and kidney stones and hypercalciuria<br>N1=56 with hPHPT<br>N2=35 with nPHPT
2	[36]	Yankova	2024	Retrospective	N=316 consecutive patients with PHPT<br>N1=266 with hPHPT<br>F:M=234:32 (Mean age=59.0 ± 11.8 y)<br>N2=48 with nPHPT<br>F:M=42:6 (Mean age= 56.9 ± 13.4 y)
3	[37]	Armstrong	2023	Retrospective	N=421 with PHPT referred for parathyroidectomy<br>F:M=307:114 (Mean age=65.6±12.2 y)<br>N1=340 with hPHPT<br>F:M=244:96 (Mean age=65.6±12.4 y)<br>N2=39 with nPHPT<br>F:M=32:7 (Mean age= 67.3±9.0 y)<br>N3=42 with nhPHPT<br>F:M=31:11 (Mean age=64.0±13.3 y)
4	[38]	Chertok Shacham	2023	Prospective, observational	N=105 referred for PHPT, osteoporosis, or elevated PTH level with normal serum calcium<br>F:M=98:7 (Mean age=69±7.9 y)<br>N1=30 with hPHPT<br>F:M=27:3 (Mean age=69.3±9.1 y)<br>N2=30 with nPHPT<br>F:M=28:2 (Mean age=69.7±7.2 y)<br>N3=45 with osteoporosis without PHPT<br>F:M=43:2 (Mean age=68.4±7.7 y)
5	[39]	Koumakis	2023	Longitudinal retrospective cohort	N=109 with PHPT and osteoporosis who underwent parathyroidectomy<br>F:M=97:12 [Median age=68 (26-92) y]<br>N1=32 with hPHPT<br>F:M=29:3 [Median age=69 (26-84) y]<br>N2=39 with nPHPT with elevated ionized calcium<br>F:M=34:5 [Median age=69 (43-92) y]<br>N3=38 with nPHPT with normal ionized calcium<br>F:M=34:4 [Median age=65 (51-85) y]
6	[40]	Tabacco	2023	Case-control	N=170 with PHPT and controls<br>F:M=159:11 (Mean age=64.9 ± 9.3 y)<br>N1=50 with hPHPT<br>F:M=47:3 (Mean age=65.2 ± 11.6 y)<br>N2=40 with nPHPT<br>F:M=37:3 (Mean age=63.4 ± 9.0 y)<br>N3=80 age-matched controls<br>F:M=75:5 (Mean age=65.4 ± 7.8 y)
7	[41]	Choi	2022	Cross-sectional	N=280 (with indication for parathyroidectomy)<br>N1=158 with hPHPT<br>F:M=120:38 (Mean age=59.3±14.0 y)<br>N2=122 with nPHPT<br>F:M=105:17 (Mean age=54.3±13.1 y)<br>N3=95 with elevated ionized Ca in nPHPT<br>F:M=82:13 (Mean age=54.2±12.1 y)<br>N4=27 with normal ionized Ca in nPHPT<br>F:M=23:4 (Mean age=54.6±16.3 y)
8	[42]	Osorio-Silla	2022	Prospective	N=87 with PHPT referred for parathyroidectomy (at the indication of an endocrinologist) (30 patients were lost to follow-up)<br>F:M=68:19<br>N1=71 with hPHPT (28 patients were lost to follow-up)<br>F:M=55:16 (Mean age=61.4±11 y)<br>N2=16 with nPHPT (2 lost to follow-up)<br>F:M=13:3 (Mean age=61.6±11 y)
9	[43]	Gomez-Ramírez	2020	Comparative	N=104 with PHPT who underwent parathyroidectomy<br>N1=88 with hPHPT<br>F:M=68:20 (Mean age=60.6±11 y)<br>N2=16 with nPHPT<br>F:M=13:3 (Mean age=60.9±10.4 y)

10	[44]	Kontogeorgos	2020	Prospective	N=750 men (population sample)<br>Age=50y<br>N1=3 with hPHPT<br>N2=21 with nPHPT<br>N3=3 with secondary HPT<br>N4=680 with normal PTH<br>N5=312 with normal calcium, PTH and vitamin D
11	[45]	Liu	2020	Observational	N=43 women with PHPT<br>N1=29 with hPHPT (Mean age=6.9±7.3 y)<br>N2=7 with nPHPT (Mean age=66.7±6.2 y)<br>N3=7 controls (Mean age=61.6±5.6 y)
12	[46]	Palermo	2020	Multicenter cross-sectional	N=127 with PHPT and controls<br>F:M=115:12 (Mean age=64.1±9.6 y)<br>N1=41 with hPHPT<br>F:M=38:3 (Mean age=63.9±12 y)<br>N2=47 with nPHPT<br>F:M=43:4 (Mean age=63.8±9.3 y)<br>N3=39 controls<br>F:M=35:4 (Mean age=64.7±7 y)
13	[47]	Schini	2020	Retrospective	N=6280 referred for BMD measurements, out of which:<br>N1=17 with hPHPT<br>F:M=15:2 (Mean age=67±6 y)<br>N2=11 with nPHPT<br>F:M=10:1 (Mean age=68±11 y)<br>N3=300 controls<br>F:M=214:86 (Mean age=70±20 y)
14	[48]	Voss	2020	Case-control	N=40 postmenopausal women with PHPT and controls<br>N1=7 with hPHPT (Mean age= 57.71±13.24 y)<br>N2=13 with nPHPT (Mean age=65.77±12.74 y)<br>N3=7 controls for N1 (Mean age=57.00±13.10 y)<br>N4=13 controls for N2 (Mean age=65.46±12.83 y)
